# Transcriptomic analysis of the physiological responses to injuries induced accompanying intracortical microelectrode implantation

**DOI:** 10.1016/j.biomaterials.2025.123692

**Published:** 2025-09-12

**Authors:** Johnathan R.T. Huff, Jaime Wang, Yue Gao, Zeynep E. Yayci, E. Ricky Chan, Allison Hess-Dunning, Jeffrey R. Capadona

**Affiliations:** a Department of Biomedical Engineering, Case Western Reserve University, 10900 Euclid Avenue, Cleveland, OH, 44106, United States; b Advanced Platform Technology Center, Louis Stokes Cleveland Veterans Affairs Medical Center, 10901 East Blvd., Cleveland, OH, 44106, United States; c Cleveland Institute for Computational Biology, Case Western Reserve University, 2109 Adelbert Road, Cleveland, OH, 44106, United States

## Abstract

The neuroinflammatory response to intracortical microelectrodes (IMEs) is regarded as a major contributor to device performance degradation and failure. Iatrogenic injuries from the craniotomy, IME insertion, and the continued presence of the implanted device all evoke a temporally mediated neuroinflammatory response. Transcriptomic analyses have emerged as a highly sensitive and detailed approach to characterizing and quantifying the neuroinflammatory response to IME implantation. Therefore, we used transcriptomic analyses to update the historic characterization of the component injuries associated with IME devices to differentiate surgical and device-related contributions to neuroinflammation and provide an updated view of a decades-old problem. Four injury profiles – uninjured, craniotomy-only, stab wound, and implant – were applied to each rat in different locations corresponding to traditional IME placement. At 2, 8, or 16 weeks after surgery, tissue was extracted and processed using bulk transcriptomic analysis with a custom panel of 212 genes related to neuroinflammation and neuronal health. Many genes were significantly differentially expressed compared to naïve controls across time points, with highly similar gene and pathway profiles observed across the craniotomy-only, stab wound, and implant groups. Additionally, the uninjured tissue region also exhibited neuroinflammation-related gene expression changes. The results presented here suggest that the neuroinflammatory response due to the intracortical implant can extend to distal brain regions. The persistent and widespread neuroinflammatory response indicates a need for novel strategies to minimize the iatrogenic effects of neural implants and suggests that the placement of additional implants for increased tissue access may come at the cost of exacerbating neuroinflammation.

## 1. Introduction

Intracortical microelectrodes (IMEs) implanted in the cortex of the brain can capture electrical signals from neurons within 50–150 μm radius of the electrode site [1,2]). The signals can be used to increase the basic understanding of the brain in health and disease, and can also be processed and translated to enable users to control computerized technologies or restore controlled movement for individuals with several motor disabilities [3-7]. Unfortunately, the clinical and experimental utility of IMEs is constrained by the progressive degradation of recorded signals over time. The neuroinflammatory response to the implanted device is regarded as a primary contributor to failure, resulting in glial encapsulation and neuronal degeneration at the implant-tissue interface [8-19].

A typical IME implant surgery involves a craniotomy to provide access to the cortex, resection of the meningeal layer(s), and insertion of the IME into cortical tissue, followed by securing the IME for chronic recording applications [20-23]. While many studies focus on the effects of the implant design on the neuroinflammatory response, the iatrogenic injuries required for placing the IMEs can also contribute to the tissue response. A craniotomy alone has been shown to disrupt the blood-brain barrier, produce inflammatory markers, and have an impact on neurological function [24-29]. Our lab and others have studied neuro- inflammatory processes elicited by the stab wound created by inserting the IME [11,18]. McConnell et al. found no significant signs of chronic inflammation by 16-weeks post-implantation as measured by immuno-histochemistry (IHC) stains for macrophages, activated microglia, astrocytes, and neuronal viability [18]. In contrast, Potter et al. reported a phasic response post-stab wound injury, with IHC markers of neuroinflammation and neuron density (*e.g.,* glial fibrillary acidic protein (GFAP) and neuronal nuclei (NeuN)) worsening for the first 8 weeks before improving, though baseline levels were not achieved by the 16-week time point [11].

IHC and similar histological techniques that allow for measuring a few (*e.g.,* 3 to 8) markers of the tissue response per tissue section have been invaluable to establishing the current understanding of the inflammatory cascade in response to IME devices and component injuries [11,12,15-19]. Among histological markers, cluster of differentiation 68 (CD68) [12,15,18,30], has been utilized to quantify the presence of activated microglia and macrophages at the site to characterize their recruitment. GFAP has been useful in characterizing the recruitment and proliferation of astrocytes characteristic of the formation of the glial scar [12,15,18,31,32]. NeuN has been the primary marker used to quantify neural cell populations and subsequent neural loss [15,18,30-32]. However, several labs have recently leveraged gene analysis techniques to study the tissue surrounding IMEs, enhancing our understanding of the neuroinflammatory response and its temporal dynamics [33-38]. Transcriptomic studies ranging from selective panels of inflammatory-related genes to evaluating the entire transcriptome have identified hundreds of genes that are involved in neuroinflammatory processes to IMEs [35,36]. However, to our knowledge, none of these studies have examined the iatrogenic injuries required for device implantation as independent events.

Here, we sought to more robustly characterize the neuro-inflammatory responses resulting from each component of the implantation procedure. We investigated the differential effects of the initial craniotomy, the injury associated with implantation (stab wound injuries), and the persistent device presence with the objective of identifying which gene pathways can be targeted to improve device integration and promote post-surgical wound healing. In this study, we used a custom panel of 212 genes related to neuroinflammation to characterize each response after 2 weeks (2WK), 8 weeks (8WK), and 16 weeks (16WK). This study provides a comprehensive characterization of the neuroinflammatory response to craniotomy, stab wounds, and implanted IMEs, offering insights that may guide future advancements in IME design and surgical techniques to optimize brain machine interface (BMI) performance.

## 2. Experimental design

**Fig. 1.**
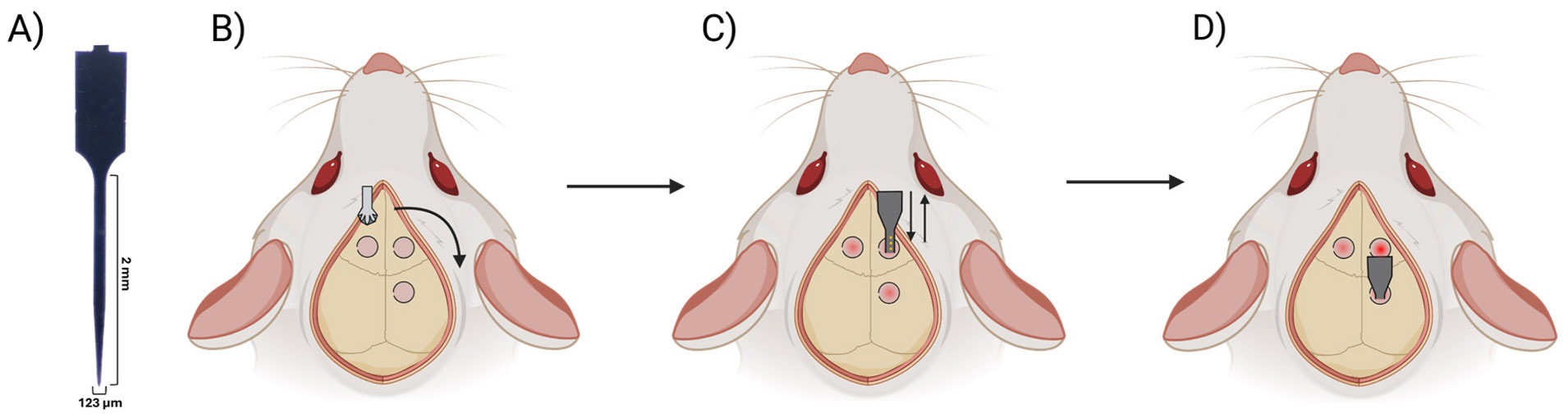
Photo of a silicon shank and graphical depiction of the process to induce the desired injuries. A) Photo of a silicon shank with shank length and width labeled B) The process begins with drilling a hole in the skull to perform a craniotomy at each injury site. C) At the second site, a single-shank silicon neural probe is inserted into the brain, then immediately removed to create a stab wound. D) The third site received a nonfunctional, single-shank silicon implant that remained in place for 2, 8, or 16 weeks. Note, each animal received the same four conditions, but the location of each condition was rotated from animal to animal to account for location-dependent effect, if any. Created in BioRender. Capadona, J. (2025) https://BioRender.com/0chl2i4.

A protocol was implemented to study the effects of each component injury associated with chronic IME devices: the craniotomy needed to access the cortex, the stab wound resulting from IME device insertion, and the continued presence of the implanted IME device. Sprague Dawley rats underwent surgery in which a different injury profile was applied to each of four quadrants of the brain, divided by the sagittal and coronal sutures (Fig. 1). One quadrant received a craniotomy (referred to as “craniotomy-only”, Fig. 1B). The next quadrant received a craniotomy and the implantation of a silicon shank (Fig. 1A), serving as an analog for a Michigan-style IME device, for 5 s before being removed (referred to as “stab wound”, Fig. 1C). The third quadrant received a craniotomy and the implantation of a silicon shank, which remained implanted (referred to as “implant”, Fig. 1D). The fourth quadrant was left undisturbed to serve as a reference for tissue in a surgical brain that was not directly impacted by an injury (referred to as “uninjured”). The injury profiles were rotated clockwise between each animal to limit any bias due to location-dependent physiological differences in the brain. The animals survived for 2-, 8-, or 16-weeks post-surgery (N = 4 per time point), at which point the animal was sacrificed via cardiac perfusion, and tissue was extracted for bulk gene expression analysis from each quadrant. Naïve rats that did not undergo a surgical procedure provided healthy control tissue that served as the baseline for gene expression analyses.

## 3. Methods

### 3.1. Intracortical implants

The implant and stab wound conditions were generated using custom-made silicon single shanks (2 mm long, 123 μm-wide, 15 μm-thick, Fig. 1A) from Qualia Labs (Dallas, TX, USA). The silicon shanks match the size and material properties of single-shank Michigan-style microelectrodes that are a gold standard in intracortical rodent recording studies. The silicon shanks were sterilized prior to surgery using ethylene oxide gas at 12.44 °C, with 1 h sterile time and 12 h aerate, and remained in a sealed container until they were used in surgeries.

### 3.2. Surgical and handling procedures

Surgical and handling procedures were based on previously established protocols [22,39] and approved by the Institutional Animal Care and Use Committee at Case Western Reserve University. A total of 16 male Sprague–Dawley rats (Charles River Labs, Wilmington, MA, USA) were utilized in the study with 4 rats serving as naïve controls and 12 rats in the experimental groups for a duration of 2 weeks (N = 4), 8 weeks (N = 4), or 16 weeks (N = 4) post-surgery. Each rat received 3 different injury profiles in 3 separate quadrants: craniotomy-only, stab wound, or implant, with a final quadrant being left uninjured. Due to an error in the RNA extraction process, one of the 8WK animals had abnormally low RNA counts and was found to be a statistical outlier when compared to the rest of the animals in terms of normalized gene expression, as indicated through a principal component analysis (PCA) in Supplemental Fig. S2. Therefore, data from that animal was excluded from the study. As a result, for all experimental groups at the 8WK time point, N = 3. For transparency, the full data set, including the outlier (indicated with a gray background) is available in Supplemental Table S3 (normalized counts) and Table S4 (raw counts).

Rats were initially anesthetized with 3.5–4.0 % isoflurane, then placed on a warming pad (PhysioSuite^®^, Kent Scientific Corporation, Torrington, CT, USA) and secured within a calibrated digital stereotaxic frame (Model 940, David Kopf Instruments, Tujunga, LA, USA) where they received a continuous flow of isoflurane at 1.5 L/min O_2_ via a nose cone. The isoflurane levels were adjusted between 1.5 % and 2.5 % to maintain a surgical plane throughout the procedure. The fur on the scalp was shaved, the toenails were clipped, and Systane eye ointment (Catalog #02444062, Alcon Canada Inc., Mississauga, ON, Canada) was applied prior to surgery site preparation. The surgical site was prepared by scrubbing with betadine followed by 70 % isopropanol in three repetitions of each. Lidocaine (10 mg/kg) was administered subcutaneously to the incision site and buprenorphine (0.05 mg/kg) was administered subcutaneously on the back prior to starting the surgery.

A 2 cm midline incision was made on the top of the head followed by a retraction of the skin and removal of the periosteum to expose the skull. Hydrogen peroxide was applied to dry the skull surface and expose the bregma and lambda landmark points for craniotomies. The exposed skull surface was primed with a drop of Vetbond tissue adhesive (Catalog #70200742529, 3 M, Saint Paul, MN, USA) to enhance dental cement adhesion to the skull.

Craniotomies were performed 2 mm laterally and 2 mm posterior/anterior in three quadrants relative to the midline and bregma (Fig. 1) using a stereotaxic frame-mounted dental drill (Catalog #MH-170, Blackstone Industries, Bethel, CT, USA), with a 1.75 mm drill bit (Catalog #514556, Stoelting Co., Wood Dale, IL, USA). The first quadrant was subjected to only a craniotomy, without further surgical intervention. The drill was set to 12,000–15,000 rpm and manually lowered to the skull. The drill was pulsed twice before fully drilling through the skull and exposing the dura [24,40]. Saline was applied to help cooling and wash out bone fragments that remained at the drill site. The exposed dura was carefully pierced and reflected using a micro point dura pick (Catalog # 10065-15, Fine Science Tools, Foster City, CA, USA), being cautious to avoid damage to the surface of the brain. The second quadrant was subjected to craniotomy and a stab wound resulting from a shank insertion through the craniotomy. Following craniotomy and dura removal, a silicon shank was inserted at 0.5 mm/s into the cerebral cortex to a depth of 1.5 mm using a NeuralGlider inserter (Actuated Medical, Bellefonte, PA, USA), then withdrawn at a rate of 0.5 mm/s to simulate the stab wound received from implantation. The third quadrant was subjected to silicon shank insertion via the same procedure but was left in place for 2, 8, or 16 weeks. Kwik-Cast silicone adhesive (World Precision Instruments, Sarasota, FL, USA) was applied to seal all three craniotomies. Additionally, the implant in the third quadrant was secured by forming a head cap with Maxcem Elite^™^ universal resin cement (Catalog #34418, KaVo Kerr, Berea, CA, USA). On both ends of the incision, the skin was closed around the head cap using 5-0 monofilament polypropylene sutures (Catalog #VCP495G, Ethicon, Raritan, NJ, USA) to promote proper healing. A single dose of cefazolin (5 mg/kg) was administered immediately post-surgery, followed by continuous administration of trimethoprim sulfamethoxazole (53 mg/kg/24 h) in the animal’s drinking water for 7 days. Subcutaneous administration of buprenorphine (0.05 mg/kg) was administered twice daily for up to 72 h for pain control. The sutures were removed 14 days post-surgery.

### 3.3. Tissue extraction and RNA isolation

Animals were sacrificed at their predetermined time points via cardiac perfusion. Prior to perfusion, the rats were anesthetized with a mixture of ketamine (100 mg/kg) and xylazine (20 mg/kg) via an intraperitoneal injection which was confirmed via toe pinch. Incisions were made through the diaphragm and rib cage to expose the heart while it was still beating. The bottom of the left ventricle was snipped and a gavage needle, attached to a peristaltic pump, was inserted up to the aorta and held in place using a hemostat. The pump was turned on, and the right ventricle was cut to relieve pressure and allow outflow. A 250–300 ml volume of 1X phosphate-buffered saline (PBS) was circulated through the bloodstream, followed by 250–300 ml of 30 % sucrose in 1X PBS to cryoprotect the brain. The rats were decapitated, and the skin, connective tissue, dental cement headcaps, silicon shanks were removed from the skull. Brains were extracted, inserted in Optimal Cutting Temperature (OCT) compound (Catalog #25608-930, Sakura Finetek, Torrance, CA, USA), flash frozen on dry ice, then stored at −80 °C until cryo-slicing.

The block of OCT was mounted in a Leica CM1860 Cryostat (Leica Biosystems, Deer Park, IL, USA) and sliced until the surface of the brain was visible. Fourteen 100 μm-thick tissue slices were retrieved from the surface of the brain to a ~1.4 mm depth, corresponding to the implant site length. The slices were punched at each injury site using a 1 mm diameter biopsy punch (Catalog #12-460-402, Fisher Scientific, Hampton, NH, USA, Integra Militrex Biopsy Punch w/Plunger), controlling for both the volume and the depth of tissue taken from each site. Each site’s selected tissue was centered on distinguishing marks made by the injuries (for stab wound and implant) or based on craniotomy coordinates when there were no visual indicators (uninjured and craniotomy-only). Naïve tissue was obtained using the same procedure with four sites in the brain tissue taken from areas correlating to the coordinates used for drilling craniotomies. Tissue from each site was pooled into 1.4 mm Omni nuclease-free bead tubes (Catalog #19–627, Omni International, Kennesaw, GA, USA), then stored at −80 °C until RNA isolation.

RNA isolation was performed using the QIAsymphony RNA kit (Catalog #931636, Qiagen, Hilden, Germany) according to the protocols provided by Qiagen (RNA_CT_400_V7, Qiagen, Hilden, Germany) [33,34,38,41]. The process began with the creation of a Buffer RLT solution made with 400 μL Buffer RLT Plus (Catalog #1053393, Qiagen, Hilden, Germany) per sample, 10 μL 2-Mercaptoethanol (Catalog #O3446I-100, Fisher Scientific, Hampton, NH, USA) per 1 mL Buffer RLT Plus, and 0.5 % v/v of Reagent DX (Catalog #19088, Qiagen, Hilden, Germany). Buffer RLT solution (400 μL) was added to each sample bead tube, and the tissue was homogenized via Omni Bead Ruptor 12 (Catalog #SKU 19–050A, Omni International, Kennesaw, GA, USA). Lysate was centrifuged for 3 min at 14,000 rpm, and the resulting supernatant was transferred to 1.5 mL Eppendorf tubes. Chloroform (100 μL) was added to each sample supernatant, and the sample was vortexed thoroughly for 3 s and centrifuged at 4 °C for 3 min at 14,000 rpm. The final sample was present at the surface of the solution and transferred to 2 mL conical tubes (Catalog #997102, Qiagen, Hilden, Germany). The conical tubes containing the samples were loaded into the sample drawer of the QIAsymphony SP along with the required reagents, consumables, and eluate cooling adapter and tubes. The RNA was extracted using the automated instrument with each sample being eluted at a 100 μL volume into 2 mL conical tubes (Catalog #997102, Qiagen, Hilden, Germany). An additional concentration step was performed utilizing the Qiagen RNeasy^®^ MinElute^®^ Cleanup Kit (Catalog #74204, Qiagen, Hilden, Germany) with the kit outlined protocol (HB-0486-004, Qiagen, Hilden, Germany) being followed for the procedure.

### 3.4. RNA bulk analysis

The transcriptomic analysis was performed using the NanoString nCounter MAX/FLEX system (Bruker Spatial Biology, Seattle, WA, United States) according to manufacturer protocols. All chemicals added to the isolated RNA for analysis were from Bruker Spatial Biology (Seattle, WA, United States). The isolated RNA from each sample was analyzed using a custom code set that targeted a panel of 218 genes, including 6 housekeeping genes and 212 genes selected for their relevance to the neuroinflammatory response using methods established in previous studies from our lab [33,34,38,41]. Capture probes were used to anchor the RNA to a plate and reporter probes containing a fluorescent barcode were read to obtain counts of genes. The custom code set was selected based on the most significantly differentially expressed genes related to neuroinflammation, oxidative stress, and neuronal health from a previous study that used a code set of 791 genes corresponding to neuroinflammation available from Bruker Spatial Biology (formerly NanoString Technologies) [33,34,38,41]. Positive and negative controls were spiked into the isolated RNA from each sample, which was then hybridized using the gene-specific capture and reporter probes for the custom panel from Bruker Spatial Biology (Seattle, WA, United States). The six positive controls are synthetic DNA targets with a set linear range of concentrations to serve as markers for hybridization efficiency. The eight negative controls are probes designed to be incompatible with hybridizing with any RNA and serve as an evaluator for non-specific probe binding rates. Tubes and reagents for the hybridization process were part of the nCounter^®^ Custom CodeSet and Prep Pack kits (Catalog #NAA-PPCK-048, Bruker Spatial Biology, Seattle, WA, United States). The hybridization process began by pipetting 70 μL of the hybridization buffer into the tube containing the Reporter CodeSet. The mixture was flick-mixed with a tube containing the Capture ProbeSet and then centrifuged before pipetting 10 μL into each tube of a 12-tube strip. Next, 5 μL of each sample and 3 μL of the Capture ProbeSet were added to each tube. Samples were incubated with the Capture ProbeSet and Reporter CodeSet for 18 h at 65 °C to allow for hybridization to occur and subsequently kept at 4 °C until samples were retrieved. The samples were loaded into the nCounter^®^ Prep Station (Catalog #NCT-PREP-LS, Bruker Spatial Biology, Seattle, WA, United States) for sample processing into cartridges. Cartridges were loaded into and analyzed by an nCounter^®^ Digital Analyzer (Catalog #NCT-DIGA-LS, Bruker Spatial Biology, Seattle, WA, United States) that scanned and counted fluorescent reporter probes and provided raw gene counts for each sample.

### 3.5. Statistical analysis

Gene count data was processed using nSolver 4.0 software (Bruker Spatial Biology, Seattle, WA, United States) as previously described [33, 34,38,41]. Background thresholding and filtering were performed based on negative control probe counts. Genes with any individual count *<*20 were replaced with a floor value of 20 prior to log2-fold change calculation to reduce the impact of near-background values. Raw counts were normalized using both positive control counts and housekeeping gene counts. Positive control normalization was performed by first calculating the global arithmetic mean across all samples of the geometric mean (geomean) of each sample’s positive controls. For each sample, the global arithmetic mean was divided by the sample geomean to obtain a sample-specific normalization factor. The raw gene counts in each sample were multiplied by the normalization factor to obtain normalized gene counts that account for differences in hybridization efficiency. Code set content normalization using housekeeping genes was performed alongside positive control normalization to account for sample-to-sample variation in RNA abundance. The six housekeeping genes were selected based on their relatively constant expression in most cells, and included hypoxanthine phosphoribosyltransferase 1 (*Hprt1*), ribosomal protein L13a (*Rpl13a*), ribosomal protein S18 (*Rps18*), succinate dehydrogenase complex flavoprotein subunit A (*Sdha*), TATA-box binding protein (*Tbp*), and ubiquitin C (*Ubc*). As with the positive control normalization, the code set content normalization was performed by multiplying a normalization factor by the gene count for each sample. The normalization factor was the geomean of the housekeeping genes for each sample divided by the arithmetic mean of the geomean of the housekeeping genes for all samples. Multiplying the raw gene counts by both the normalization factor for the positive controls and the housekeeping genes generated the normalized gene counts. Normalized counts were processed using nSolver Advanced Analysis 2.0 to determine differential expression using a simplified negative binomial model to generate log2-fold changes and p-values. Genes with low abundance across samples were excluded from an individual differential analysis and labeled low counts (LC) in Table S1 if more than 75 % of their counts were ≤20, consistent with background levels. The Benjamini-Hochberg correction factor with a false discovery rate of 0.2 was used to reduce potential false positive genes due to multiple testing and adjusted p-values (p_ad_j) were calculated. All genes with a p_ad_j *<* 0.05 were considered significant. A log2-fold change cutoff was not included when analyzing the data.

For visualization of differential gene expression profiles, GraphPad Prism 10 (GraphPad Software, Boston, Massachusetts USA) was used to generate volcano plots. Significantly differentially expressed genes (padj *<* 0.05 when comparing to non-surgical controls) were categorized into six groups designated as functional categories based on their primary function and the types of cells in which they are primarily expressed: inflammation, immune cells, oxidative stress, apoptosis, neural health, and homeostasis/growth. The functional categories were determined and assigned based on a literature search on the functions of each gene contained in the 212 gene panel. The design of the six functional categories was a balance of conveying important information that could be broken down in figures while avoiding creating too many niche categories. Supplemental Table S1 lists the genes that were significantly expressed in each category, their log2-fold changes, and their adjusted p-values. Advaita iPathwayGuide was used to identify pathways involving significantly expressed genes. Gene name, log2-fold change, and padj were input into the software to generate the desired analyses. The pathway analysis utilized the Kyoto Encyclopedia of Gene and Genomes (KEGG) to organize genes expressed by each experimental group into various pathways and associated processes. Gene ontology (GO) terms were also used to identify biological processes enriched with significantly differentially expressed genes. The pathway and biological processes analysis identified which transcriptionally regulated processes related to the functional categories were affected in each experimental group.

## 4. Results

**Fig. 2.**
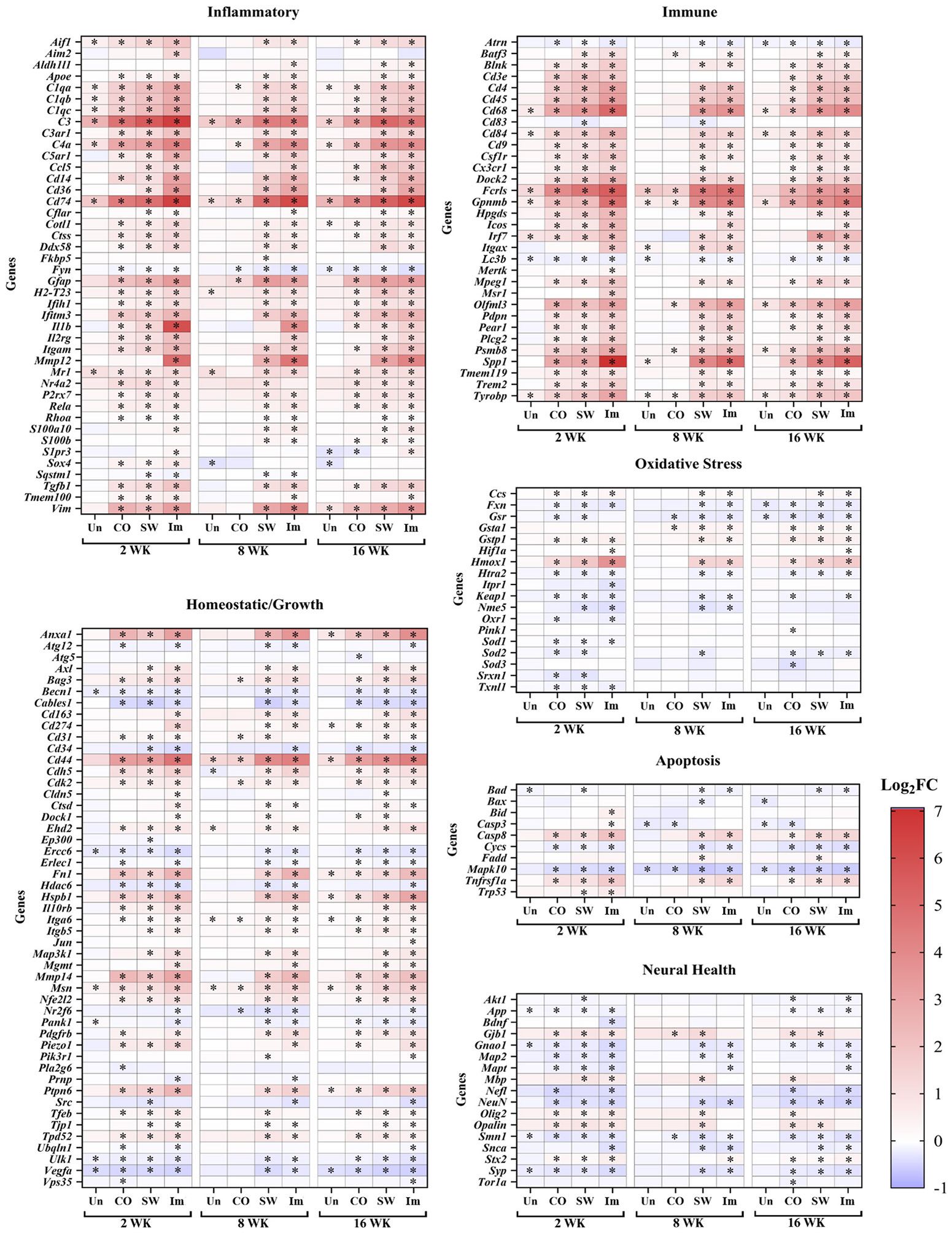
Heatmaps of differentially expressed genes in every functional category. Red represents upregulation and blue represents downregulation compared to naïve controls. Genes were included if they were differentially expressed in at least 1 experimental group. Stars are located on where genes were significantly expressed (p_adj_ < 0.05). Each experimental group was labeled using a time point: 2WK, 8WK, or 16WK. The group labeling was followed by the injury type: Un (Uninjured), CO (Craniotomy-only), SW (Stab Wound), or Im (Implant). A-F) Heatmap of genes expressed in each functional category and how they compare across injury groups and timepoints. A) Inflammatory category B) Immune category C) Homeostatic/Growth category D) Oxidative stress category E) Apoptosis category F) Neural health category.

The differential expression of 212 genes was analyzed in rats 2WK, 8WK, and 16WK post-surgery was compared to naïve control animals that provided a reference for healthy brain tissue. Each animal in the experimental groups contained four sites of interest: a site where a craniotomy was solely performed (craniotomy-only), a site with a craniotomy and a stab wound (stab wound), a site with a craniotomy and an indwelling silicon shank implant (implant), and a site receiving no injury (uninjured). Of the 212 genes related to neuroinflammation in the custom panel, 168 genes were significantly differentially expressed in at least one of the groups for at least one time point. Heatmaps showing the significantly differentially expressed genes for each functional category are shown in Fig. 2. The genes in the inflammation and immune functional groups are predominantly upregulated while genes in the oxidative stress, apoptosis, and neural health functional groups are predominantly downregulated, and genes in the homeostasis/ growth functional group displayed a mix of both up- and downregulation. Within each time point, the absolute value of the log2-fold change generally increases with increasing injury invasiveness, with the uninjured groups having the smallest log2-fold change compared to naïve controls and the implant group having the highest log2-fold change compared to naïve controls. However, within a treatment, there was little change in log2-fold change across time points, except for a decrease in the magnitude of the log2-fold change for the 8WK craniotomy-only group.

### 4.1. Two weeks post-surgery

**Fig. 3.**
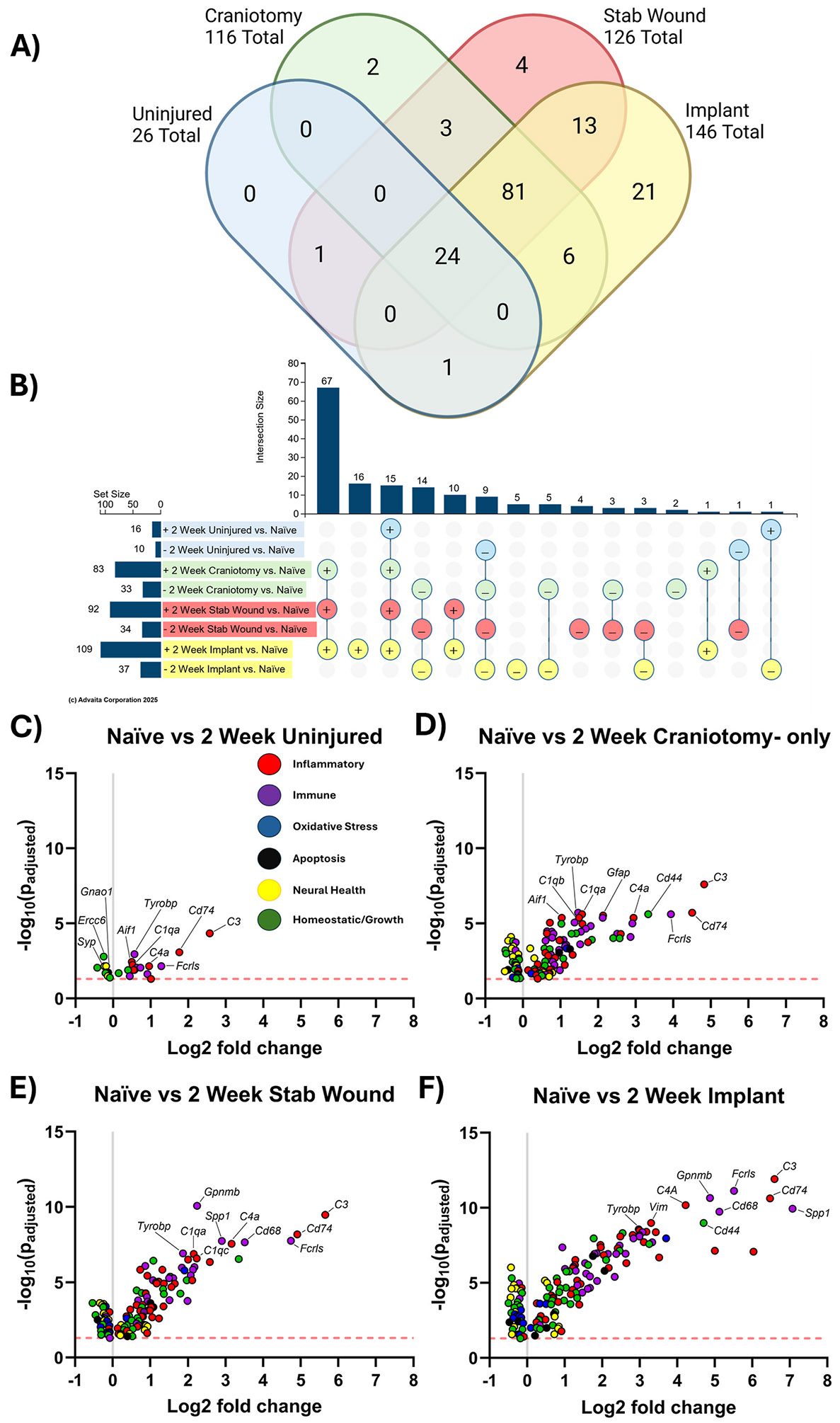
Differential expression of neuroinflammatory genes in rats 2 weeks post-surgery (n = 4 per condition) compared to non-surgical naïve rats (n = 16): A) Venn diagram containing the number of significantly (p_adj_ < 0.05) differentially expressed genes under each injury condition. The overlapping regions indicate differentially expressed genes shared among the injury conditions. The Venn diagram does not distinguish between genes that are up- or downregulated. B) UpSet plot of the significantly (p_adj_ < 0.05) differentially expressed genes under each injury condition, and how their expression overlaps. Within the plots, plus signs indicate genes that were upregulated in a group and the minus signs indicate genes that were downregulated. The connected dots under each bar indicate the overlap associated with the intersection size. C–F) Volcano plots show the differentially expressed genes for each injury condition, where each point represents a gene in the panel. The color of each point indicates the gene function, as indicated by the legend. The gray line marks the divide between up- and downregulated genes, with upregulated genes located to the right of the line and downregulated genes located to the left. The dotted red line marks the threshold (p_adj_ < 0.05) for genes to be considered significant. Genes that did not meet the significance threshold (p_adj_ < 0.05) were excluded from the volcano plots. Most differentially expressed genes are upregulated, with comparatively few downregulated genes.

In the 2W post-surgery group, all four conditions showed significant differential gene expression compared to naïve tissue, with the implant sites exhibiting the most substantial tissue response (Fig. 3). Within the panel of 212 neuroinflammation-related genes analyzed, the implant sites showed the highest differential expression with 146 genes, followed by the stab wound (126 genes), craniotomy-only sites (116 genes), and uninjured tissue (26 genes) (Fig. 3A). The Venn diagram in Fig. 3A shows substantial overlap in the significantly differentially expressed genes across conditions, with the UpSet plot in Fig. 3B providing additional context by indicating the up- and down-regulation of the differentially expressed genes. The implant site shared 25 out of 26 (96 %, 15 upregulated, 9 downregulated, 1 mixed) differentially expressed genes from the uninjured condition, 111 out of 116 (96 %, 83 upregulated, 28 downregulated) genes from the craniotomy-only condition, and 118 out of 126 (~94 %, 92 upregulated, 26 downregulated) genes from the stab wound condition. Roughly 68 % (105 of 154) of the differentially expressed genes in the craniotomy, stab wound, or implant sites were shared between all three groups with ~76 % (82 of 108) of the significantly upregulated, and ~51 % (23 of 45) of significantly down- regulated genes being shared. Except for a single gene, pantothenate kinase 1(*Pank1*), genes that were differentially expressed in multiple groups showed the same direction of change across groups with significant differential expression. *Pank1*, which is involved in homeostatic processes through its role in synthesis of coenzyme A (CoA) [42], displayed mixed regulation as it was upregulated in the uninjured group and downregulated in the implant group. Twenty-four of 26 genes that were differentially expressed in the uninjured tissue were also differentially expressed in the craniotomy-only condition, demonstrating over 92 % conservation of the uninjured phenotype in the craniotomy-only condition. When adding the second injury of stab wound to the craniotomy injury, 108 of the 116 (93 %) genes remained differentially expressed, with 10 (~9 %) genes losing significance, and 18 genes becoming newly significantly differentially expressed. Those 18 genes represented ~14 % of the significantly differentially expressed genes (18 of 126) in the stab wound group. Likewise, 118 of 126 (~94 %) genes that were differentially expressed in the stab wound group remained differentially expressed when the implant remained in the tissue. Furthermore, 28 additional genes became significantly differentially expressed in the implant group that were not expressed in the stab wound group, representing ~19 % of the 146 differentially expressed genes found at the implant site. Across all conditions, 2 genes, both downregulated, were uniquely differentially expressed in the craniotomy-only condition, 4 genes, all downregulated, were uniquely differentially expressed in the stab wound condition, 21 (16 upregu- lated, 5 downregulated) genes were uniquely differentially expressed in the implant condition, and no genes were uniquely differentially expressed in the uninjured condition. The volcano plots in Fig. 3C-F show the differential expression profiles for each injury site compared to the naïve tissue, with log2-fold change >0 corresponding to upregulated genes and log2-fold change <0 corresponding to downregulated genes. The plots show that the number of differentially expressed genes and average log2-fold change increase with increased procedure invasiveness. The uninjured site had the lowest average log2-fold change compared to the naïve control, and the implant site had the highest.

**Fig. 4.**
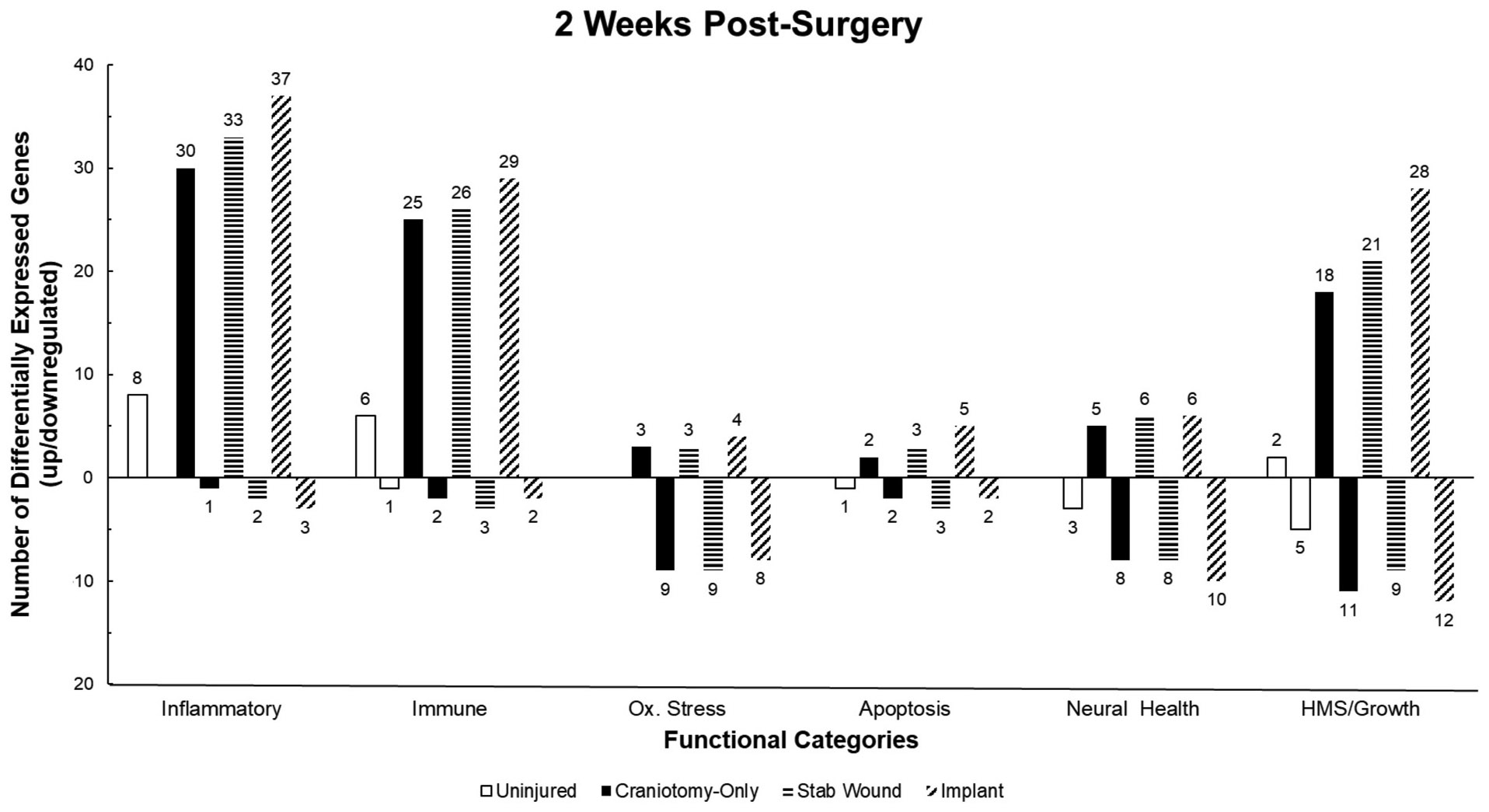
Bar graph showing the gene expression functional category breakdown at 2 weeks post-surgery. Counts represent the number of differentially expressed genes for each injury profile and functional category. Counts above the x-axis are genes that are significantly upregulated and counts below the x-axis are significantly downregulated compared to naïve sham controls.

Fig. 4 summarizes the upregulation and downregulation of differentially expressed genes associated with inflammation, immune response, oxidative stress, apoptosis, neural health, homeostasis/growth for each injury profile. All injured sites had extensive inflammation-related differentially expressed genes with ≥30 upregulated and ≤3 downregulated inflammatory genes, and ≥25 upregulated with ≤3 downregulated immune genes. Fig. 2A and B shows the majority of genes in both inflammatory and immune categories are significantly expressed with genes such as complement component 3 (*C3*), glial fibrillary acidic protein (*Gfap*), cluster of differentiation 68 (*Cd68*), and glycoprotein nmb (*Gpnmb*) maintaining high, upregulated expression and FYN proto-oncogene, src family tyrosine kinase (*Fyn*), microtubule associated protein 1 light chain 3 beta (*Lc3b*) and attractin (*Atrn*) maintaining downregulation. Absent in melanoma 2 (*Aim2*), matrix metallopeptidase 12 (*Mmp12*), and macrophage scavenger receptor 1 (*Msr1*) were unique in their increase in expression, making sudden jumps from little to no expression at the acute injury sites to high, significant expression at the implant site. *Aim2* produces a protein that creates the AIM2 inflammasome complex, which acts as a trigger for inflammation; *Mmp12* encodes a protein primarily expressed by macrophages that is responsible for extracellular membrane breakdown, and *Msr1* is an indicator for proinflammatory macrophage activation [43-45].

Oxidative stress and neural health differentially expressed genes were mostly downregulated across the injured sites, with each group having 3 or 4 upregulated and 8 or 9 downregulated genes in the oxidative stress category, and 5 or 6 genes upregulated with 8–10 genes downregulated in the neural health category. Fig. 2D and F shows the genes in the oxidative stress category having a little more variability with the neural health category largely overlap in expression. Differentially expressed genes in the oxidative stress category that were consistently expressed included upregulated heme oxygenase 1 (*Hmox1*), glutathione s-transferase pi 1 (*Gstp1*), and copper chaperone for superoxide dismutase (*Ccs*) and downregulated frataxin (*Fxn*), HtrA serine peptidase 2 (*Htra2*), kelch-like ECH associated protein 1 (*Keap1*), superoxide dismutase 1 (*Sod1*), and thioredoxin like 1 (*Txnl1*). Differentially expressed genes in the neural health category such as oligo-dendrocytic myelin paranodal and inner loop protein (*Opalin*), oligodendrocyte transcription factor 2 (*Olig2*), and gap junction protein beta 1 (*Gjb1*) maintained upregulation, while RNA binding fox-1 homolog 3 (*NeuN*), Synaptophysin (*Syp*), G protein subunit alpha o1 and (*Gnao1*) were downregulated among injured sites.

The injured sites had mixed differential expression of apoptosis-related genes with 2–5 upregulated and 2 or 3 downregulated genes. Fig. 2E shows Caspase 8 (*Casp8*) and TNF receptor superfamily member 1a (*Tnfrsf1a*) maintain upregulated expression and mitogen-activated protein kinase 10 (*Mapk10*) and cytochrome *c*, somatic (*Cycs*) down-regulated in their expression across injured sites. In the homeostatic/growth category, a majority (≥18) of the differentially expressed genes were upregulated for all injured sites, but there was also a large number of downregulated genes (≥9). Fig. 2C shows a majority of differentially expressed genes are overlapping with genes such as annexin a1 (*Anxa1*), cluster of differentiation 44 (*Cd44*), and matrix metallopeptidase 14 (*Mmp14*) maintaining high amounts of upregulated expression and genes such as cdk5 and abl enzyme substrate 1 (*Cables1*), excision repair 6, chromatin remodeling factor (*Ercc6*), and vascular endothelial growth factor a (*Vegfa*) having consistent downregulation. Overall, the craniotomy-only site had 83 upregulated and 33 downregulated differentially expressed genes; the stab wound site had 92 upregulated and 34 downregulated differentially expressed genes; and the implant site had 109 upregulated and 37 downregulated differentially expressed genes.

The uninjured site had similar ratios of significantly differentially expressed upregulated vs. downregulated genes, though the overall numbers were smaller with 8 upregulated inflammatory genes, 6 upregulated immune genes, and a single downregulated immune gene. No oxidative stress genes and 1 downregulated apoptosis gene were differentially expressed. The uninjured site had 3 downregulated neural health genes. In the homeostatic/growth genes functional group, the uninjured site differed from the injured sites with differential expression of more downregulated [5] than upregulated [2] genes. Fig. 2 shows expression in each functional category overlaps greatly with the injured sites. At the uninjured site, a total of 16 differentially expressed genes were upregulated and 10 were downregulated.

### 4.2. Eight weeks post-surgery

**Fig. 5.**
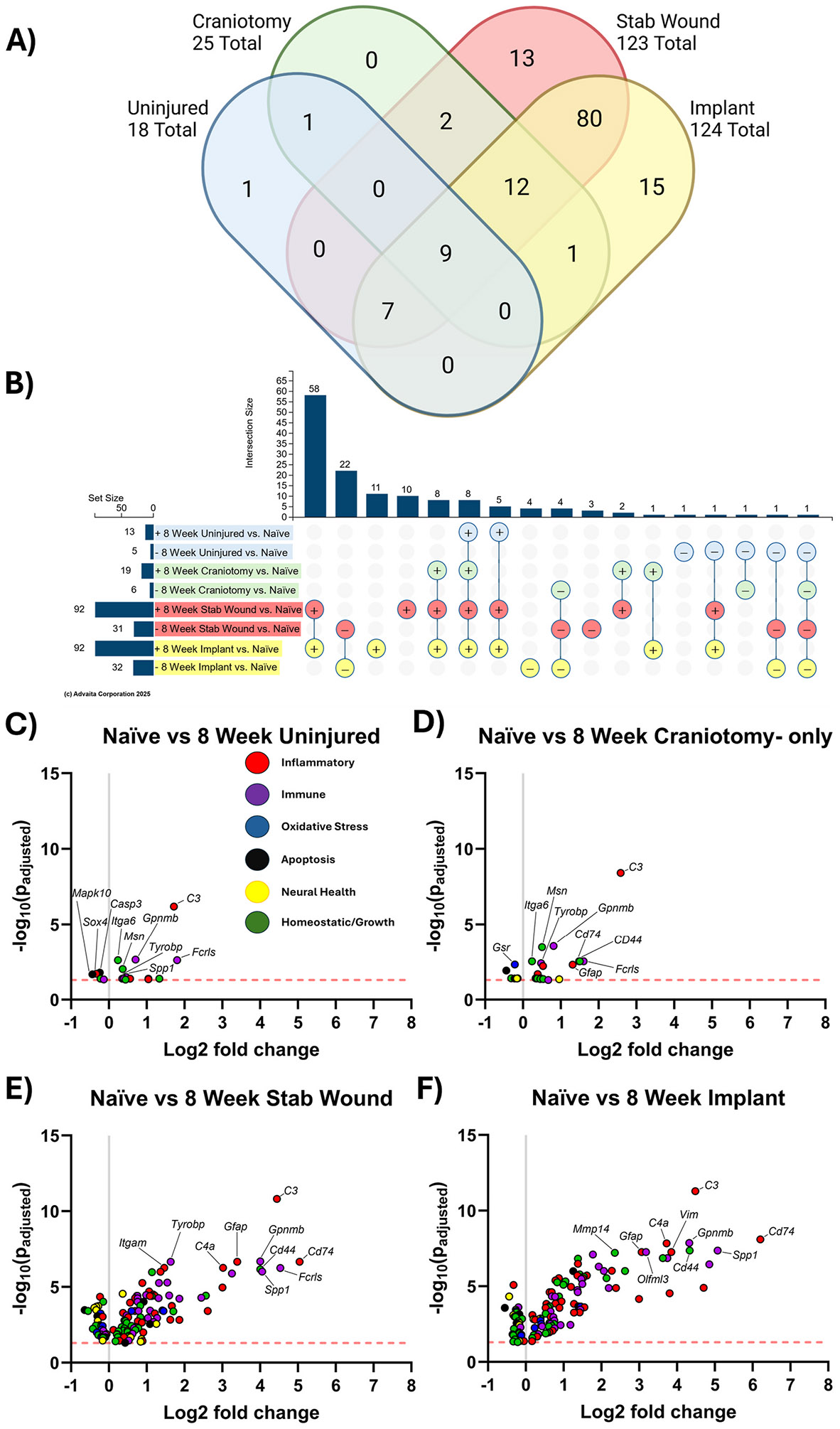
Differential expression of neuroinflammatory genes in rats 8 weeks post-surgery (n = 3 per condition) compared to non-surgical naïve rats (n = 16): A) Venn diagram containing the number of significantly (p_adj_ < 0.05) differentially expressed genes at each injury condition. The overlapping regions indicated differentially expressed genes that were shared among the injury conditions. The Venn diagram does not distinguish between genes that are up or downregulated. B)UpSet plot of the significantly (p_adj_ < 0.05) differentially expressed genes under each injury condition, and how their expression overlaps. Within the plots, plus signs indicate genes that were upregulated in a group and the minus signs indicate genes that were downregulated. The connected dots under each bar indicate the overlap associated with the intersection size. C–F) Volcano plots show the differentially expressed genes for each injury condition, where each point represents a gene in the panel. The gray line marks the divide between up- and downregulated genes with upregulated genes located to the right of the line and downregulated genes located to the left. The dotted red line marks the threshold (p_adj_ < 0.05) for genes to be considered significant. Genes that did not meet the significance threshold (p_adj_ < 0.05) were excluded from the volcano plots.

In the eight-week post-surgery group, all four conditions again showed significant differential gene expression compared to naïve tissue, with the implant sites exhibiting the most substantial tissue response (Fig. 5). Within the panel of 212 neuroinflammation-related genes analyzed, the implant sites showed the highest differential expression with 124 genes, followed by the stab wound (123 genes), craniotomy-only sites (25 genes), and uninjured tissue (18 genes) (Fig. 5A). The Venn diagram in Fig. 5A shows substantial overlap in the significantly differentially expressed genes across conditions, with the UpSet plot in Fig. 5B providing additional context by indicating the up- and downregulation of the differentially expressed genes. The implant sites shared 16 out of 18 (~89 %, 13 upregulated, 2 downregulated, 1 mixed) differentially expressed genes from the uninjured condition, 22 out of 25 (88 %, 17 upregulated, 5 downregulated) genes from the craniotomy-only condition, and 108 out of 123 (~88 %, 90 upregulated, 28 downregulated) genes from the stab wound condition. Unlike the 2-week time point, at eight weeks post-injury, ~15 % of the differentially expressed genes seen in the craniotomy, stab wound, or implant sites were shared between all three groups (21 of 141) with ~16 % of the significantly upregulated (16 of 103) and ~14 % of significantly downregulated (5 of 37) genes being shared. Except for a single gene, cadherin 5 (*Cdh5*), all genes that were differentially expressed in multiple groups showed the same direction of change across groups with significant differential expression. *Cdh5*, which is involved in maintaining or restoring blood-brain barrier (BBB) integrity, displayed mixed regulation with upregulation in the stab wound and implant groups and downregulation in the uninjured group [46]. In total, 16 of 18 genes that were differentially expressed in the uninjured tissue were also differentially expressed in the craniotomy-only condition, demonstrating over 89 % conservation of the uninjured condition phenotype in the craniotomy condition. When adding the second injury of a stab wound to the craniotomy injury, 23 of the 25 (92 %) genes remained differentially expressed, with 2 (8 %) genes losing significance. An additional 100 genes became newly significantly differentially expressed, and 108 of 123 (~88 %) genes that were differentially expressed in the stab wound group remained differentially expressed when the implant remained in the tissue. Furthermore, 16 additional genes became significantly differentially expressed in the implant group that were not expressed in the stab wound group, representing ~13 % of the 124 differentially expressed genes found at the implant site (15 of these genes were unique to the implant site). Across all conditions, no genes were uniquely differentially expressed in the craniotomy-only condition, 13 genes (10 upregulated, 3 downregulated) were uniquely differentially expressed in the stab wound condition, 15 genes (11 upregulated, 4 downregulated) were uniquely differentially expressed in the implant condition, and 1 downregulated gene was uniquely differentially expressed in the uninjured condition. The volcano plots in Fig. 5C-F show the differential expression profiles for each injury site compared to the naïve tissue. The plots show that the number of differentially expressed genes and average log2-fold change increase with increased procedure invasiveness. The uninjured site had the lowest average log2-fold change compared to the naïve control, and the implant site had the highest.

**Fig. 6.**
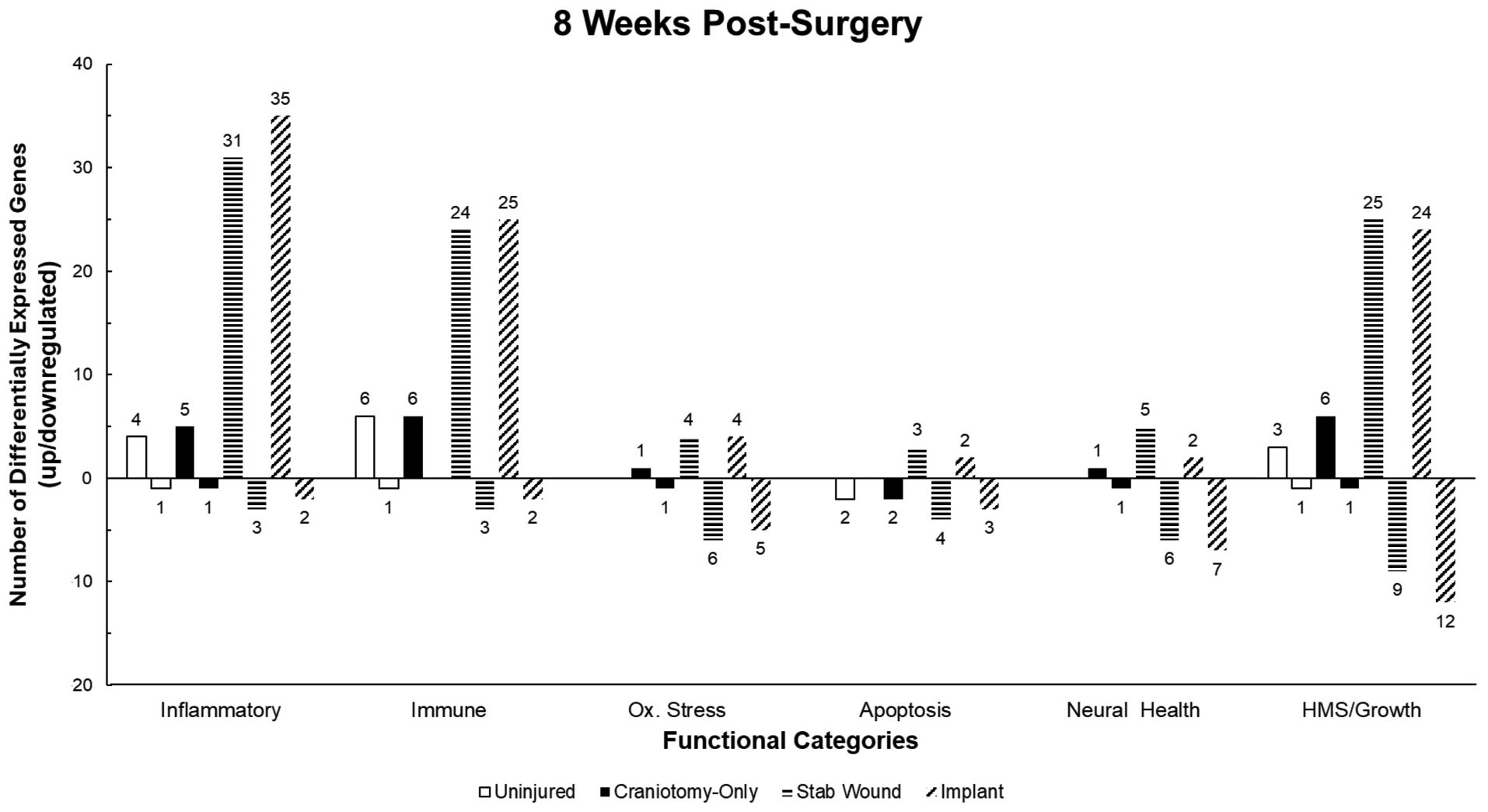
Bar graph showing the gene expression functional category breakdown at 8 weeks post-surgery. Counts represent the number of differentially expressed genes for each injury profile and functional category. Counts above the x-axis are genes that are significantly upregulated, and counts below the x-axis are significantly downregulated.

Fig. 6 summarizes the upregulation and downregulation of differentially expressed genes associated with inflammation, immune response, oxidative stress, apoptosis, neural health, and homeostasis/growth for each injury profile. The 8WK craniotomy-only group had a unique decrease in overall gene expression from the 2WK timepoint. Fig. 2 shows which genes lose significance at 8WK from 2WK and which ones maintain significant expression. Even with a large decrease in significant expression, similar patterns of expression to the other craniotomy-only groups in the study are present. The craniotomy-only site maintains a majority upregulation of inflammatory and immune differentially expressed genes, with 5 upregulated and 1 downregulated in the inflammatory category and 6 upregulated in the immune category (Fig. 2A and B). Inflammatory and immune-related differentially expressed genes that notably maintained a high amount of expression were *C3*, cluster of differentiation 74 (*Cd74*), *Gfap*, and Fc receptor like 2 (*Fcrls*). Oxidative stress and neural health genes share mixed differential expressions with both having 1 upregulated and 1 downregulated gene. Fig. 2D shows glutathione s-transferase alpha 1 (*Gsta1*), a mitigator of oxidative stress, becomes uniquely significantly upregulated from 2WK [47]. The 8WK craniotomy-only group is unique from other craniotomy-only groups in having solely downregulation of 2 apoptotic differentially expressed genes and a vast majority upregulation of 6 homeostatic/growth differentially expressed genes with 1 down-regulated. Apoptotic *Casp3*, a primary regulator of apoptosis with roles in inflammation, and homeostatic nuclear receptor subfamily 2 group f member 6 (*Nr2f6*), a transcriptional inhibitor of adaptive immune cells, in Fig. 2C and E are uniquely significantly downregulated at 8WK when they were not significantly expressed at 2WK [48-50]. The craniotomy-only site had a total of 19 upregulated and 6 downregulated differentially expressed genes.

The implant and stab wound injury groups maintained extensive inflammation-related differential gene expression with ≥31 upregulated and 2 or 3 downregulated inflammatory genes, and ≥24 upregulated with 2 or 3 downregulated immune genes. Both injured sites had most genes decrease in their inflammatory and immune expression with several losing significance as shown in Fig. 2A and B. *Aim2* and *Msr1* were no longer significantly differentially expressed at 8WK, while *Mmp12* remained differentially expressed at the implant site and became highly expressed at the stab wound site compared to 2WK. Oxidative stress and neural health differentially expressed genes continued to be mostly downregulated across the injured sites, with each group having 4 upregulated and 5 or 6 genes downregulated in the oxidative stress category, and 2 to 5 genes upregulated with 6 or 7 genes downregulated in the neural health category. Fig. 2D and F outline a reduction of differential expression in both oxidative stress and neural health categories, with the exception of newly significantly upregulated *Gsta1* at the 8WK stab wound and implant sites. Notably, the stab wound group uniquely maintained upregulation of differentially expressed genes related to myelination and oligodendrocyte function, such as *Mbp, Olig2*, and *Opalin* [51-53]. The stab wound and implant sites also maintained mixed expression of apoptotic differentially expressed genes, with 2 or 3 upregulated and 3 or 4 downregulated genes. Fig. 2E shows upregulated apoptotic genes at the implant and stab wound groups largely had a reduction in differential expression, except for the new significance for the stab wound group of Fas-associated death domain (*Fadd*), a facilitator of linking death receptors with caspases [54]. Downregulated differentially expressed genes had an increased number of downregulated genes with BCL2 associated agonist of cell death (*Bad*), a binder of anti-apoptotic factors, becoming significantly downregulated at the implant site and BCL2 associated x, apoptosis regulator (*Bax*), a mitochondrially associated apoptosis activator, becoming significantly downregulated at the stab wound site [55,56]. In the homeostatic/-growth category, a majority (≥24) of the differentially expressed genes were still upregulated for all injured sites, and many downregulated genes (≥9) were similarly maintained. Fig. 2C outlines an increase in differential expression for the stab wound site at 8WK and a small decrease at the implant site. Most differentially expressed genes overlap between the stab wound and implant sites with *Anxa1, Cd44*, and *Mmp14* still significantly upregulated and *Cables1, Ercc6*, and *Vegfa* still significantly downregulated at both stab wound and implant sites. Overall, the stab wound site had 91 upregulated and 32 downregulated differentially expressed genes, and the implant site had 93 upregulated and 31 downregulated differentially expressed genes.

The uninjured site had similar but slightly reduced inflammation-related differential expression compared to the 2WK timepoint with 4 upregulated and 1 downregulated inflammatory gene, and 6 upregulated with 1 downregulated immune gene. Histocompatibility 2, t region locus 23 (*H2-T23*), integrin subunit alpha X (*Itgax*), and secreted phosphoprotein 1 (*Spp1*) became significantly upregulated at 8WK, while SRY-box transcription factor 4 (*Sox4*) became significantly downregulated, as shown in Fig. 2A and B. No oxidative stress genes and 2 downregulated apoptosis genes were differentially expressed. Fig. 2D shows that caspase 3 (*Casp3*) and *Mapk10* became significantly down-regulated at 8WK. The uninjured site uniquely had no neural health genes differentially expressed, and flipped homeostatic/growth differential expression as it was largely downregulated at 2WK and largely upregulated at 8WK, with 3 upregulated and 1 downregulated homeostatic/growth genes. Fig. 2C shows all significantly downregulated genes at 2WK lost significance, while *Cd44*, EH domain containing 2 (*Ehd2*), and integrin subunit alpha 6 (*Itga6*) became significantly upregulated. *Cdh5* was uniquely significantly downregulated at 8WK. At the uninjured site, a total of 13 differentially expressed genes were upregulated and 5 were downregulated.

### 4.3. Sixteen weeks post-surgery

**Fig. 7.**
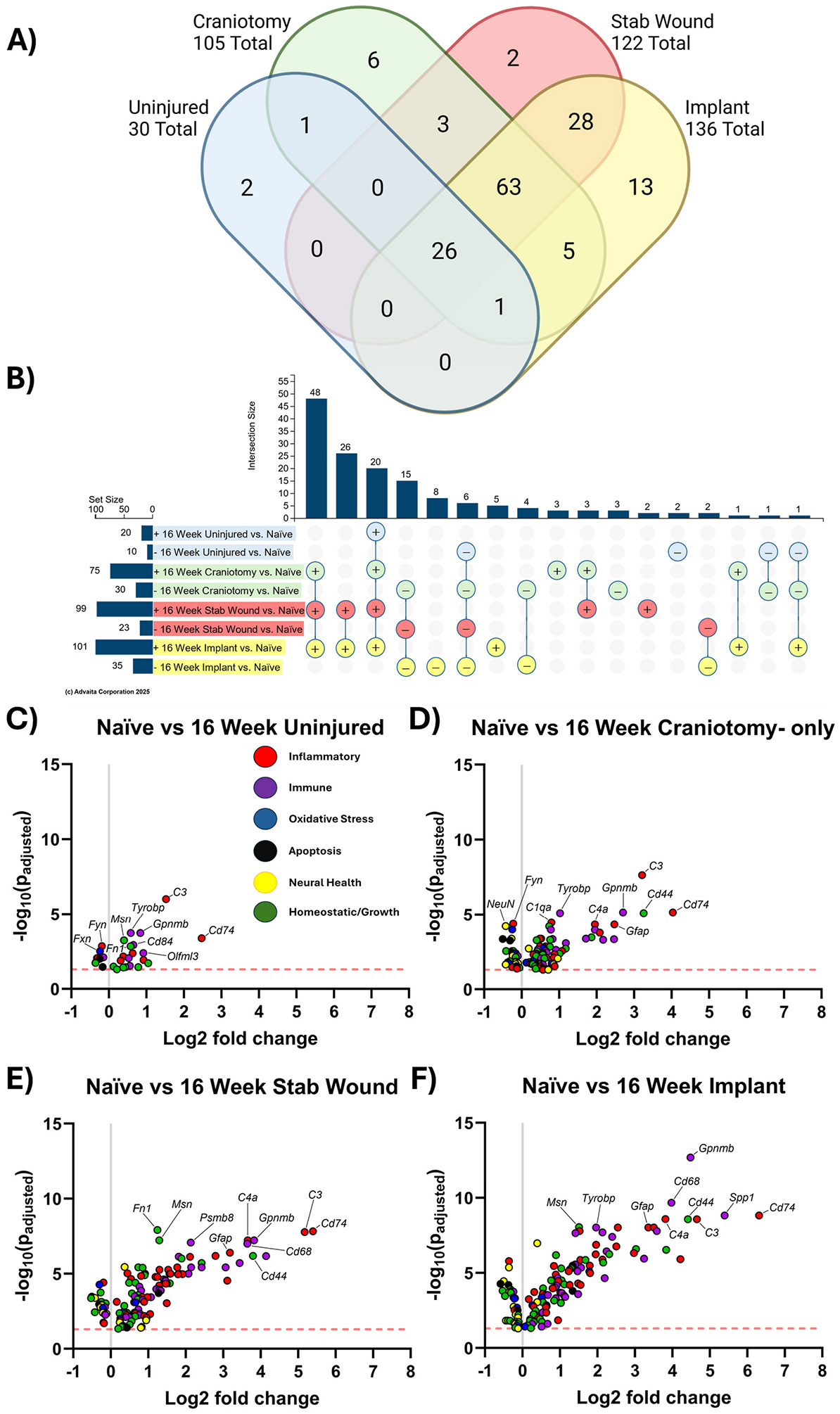
Differential expression of neuroinflammatory genes in rats 16 weeks post-surgery (n = 4 per condition) compared to non-surgical naïve rats (n = 16): A) Venn diagram containing the number of significantly (p_adj_ < 0.05) differentially expressed genes at each injury site. The overlapping regions indicated differentially expressed genes that were shared among the injuries. The Venn diagram does not distinguish between genes that are up- or downregulated. B) UpSet plot of the significantly (p_adj_ < 0.05) differentially expressed genes under each injury condition, and how their expression overlaps. Within the plots, plus signs indicate genes that were upregulated in a group and the minus signs indicate genes that were downregulated. The connected dots under each bar indicate the overlap associated with the intersection size. C–F) Volcano plots show the differentially expressed genes for each injury condition, where each point represents a gene in the panel. The gray line marks the divide between up- and downregulated genes, with upregulated genes located to the right of the line and downregulated genes located to the left. The dotted red line marks the threshold (p_adj_ < 0.05) for genes to be considered significant. Genes that did not meet the significance threshold (p_adj_ < 0.05) were excluded from the volcano plots.

In the sixteen-week post-surgery group, all four conditions showed significant differential gene expression compared to naïve tissue, with the implant sites exhibiting the most substantial tissue response (Fig. 7). Within the panel of 212 neuroinflammation-related genes analyzed, the implant sites showed the highest differential expression with 136 genes, followed by the stab wound (122 genes), craniotomy-only sites (105 genes), and uninjured tissue (30 genes) (Fig. 7A). The Venn diagram in Fig. 7A reveals substantial overlap in the significantly differentially expressed genes across conditions, with the UpSet plot in Fig. 7B providing additional context by indicating the up- and down-regulation of the differentially expressed genes. The implant sites shared 27 out of 30 (90 %, 20 upregulated, 6 downregulated, 1 mixed) differentially expressed genes from the uninjured condition, 95 out of 105 (~90 %, 68 upregulated, 26 downregulated, 1 mixed) genes from the craniotomy-only condition, and 117 out of 122 (~96 %, 94 upregulated, 23 down-regulated) genes from the stab wound condition. Approximately 59 % (89 of 150) of the differentially expressed genes in the craniotomy, stab wound, or implant sites were shared between all three groups, with ~63 % (68 of 108) of the significantly upregulated, and ~51 % (21 of 41) of the significantly downregulated genes being shared. Except for a single gene, Sphingosine-1-phosphate receptor 3 (*S1*pr*3*), all genes that were differentially expressed in multiple groups showed the same direction of change across groups with significant differential expression. *S1*pr*3* is involved in modulating the toll-like receptor 2/4(TLR2/4)-nuclear factor kappa B (NF-κB) signaling pathway, and displayed mixed regulation with upregulation in the implant group and downregulation in the uninjured and craniotomy-only group [57]. In total, 28 of 30 genes that were differentially expressed in the uninjured tissue were also differentially expressed in the craniotomy-only condition, demonstrating over 93 % conservation of the uninjured condition phenotype in the craniotomy condition. When adding the second injury of a stab wound to the craniotomy injury, 92 of the 105 (~88 %) genes remained differentially expressed, with 13 (~12 %) genes losing significance. An additional 31 genes became newly significantly differentially expressed. Likewise, 117 of 122 (~96 %) genes that were differentially expressed in the stab wound group remained differentially expressed when the implant remained in the tissue. Furthermore, 19 additional genes became significantly differentially expressed in the implant group that were not differentially expressed in the stab wound group, representing ~14 % of the 136 differentially expressed genes found at the implant site (13 of these genes were unique to the implant site). Across all conditions, 6 genes (3 upregulated, 3 downregulated) were uniquely differentially expressed in the craniotomy-only condition, 2 upregulated genes were uniquely differentially expressed in the stab wound condition, 13 genes (5 upregulated, 8 downregulated) were uniquely differentially expressed in the implant condition, and 2 downregulated genes were uniquely differentially expressed in the uninjured condition. The volcano plots in Fig. 7C-F show the differential expression profiles for each injury site compared to the naïve tissue. Fig. 7 corroborates the trends from 2WK and 8WK time points, with increased procedure invasiveness leading to an increased number of significantly expressed genes and average log2-fold change. The uninjured site had the lowest log2-fold change compared to the naïve control, and the implant site had the highest log2-fold change, as well as the most upregulated genes.

**Fig. 8.**
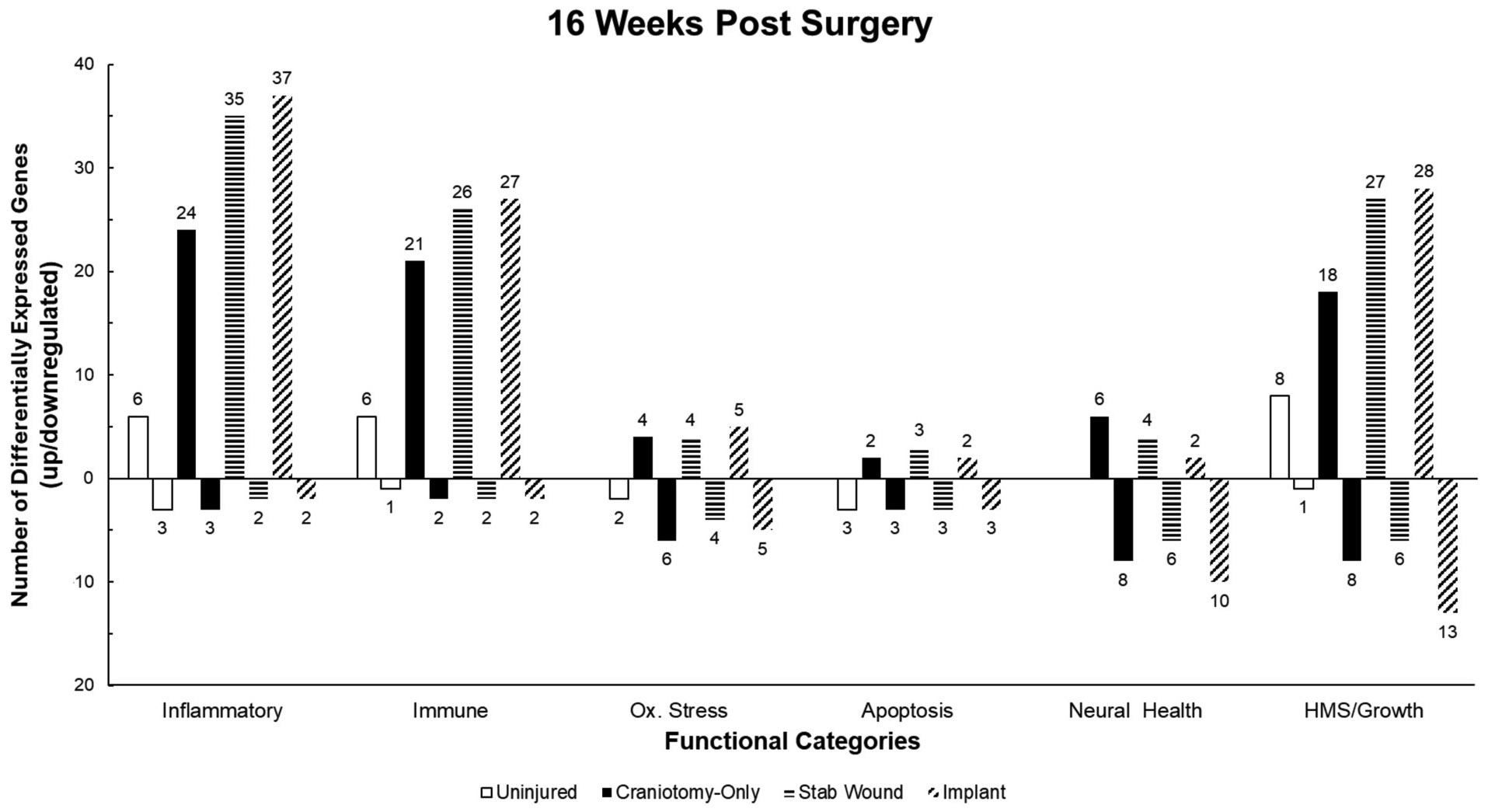
Bar graph showing the gene expression functional category breakdown at 16 weeks post-surgery. Counts represent the number of differentially expressed genes for each injury profile and functional category. Counts above the x-axis are genes that are significantly upregulated and counts below the x-axis are significantly downregulated.

Fig. 8 summarizes the upregulation and downregulation of differentially expressed genes associated with inflammation, immune response, oxidative stress, apoptosis, neural health, and homeostasis/growth for each injury profile. The 16WK craniotomy-only group had a large increase in several functional categories from the 8WK craniotomy-only group and returned to a pattern highly similar to the 2WK craniotomy-only group. Fig. 2 shows every functional category except apoptosis gaining many differentially expressed genes at 16WK compared to 8WK. The other experimental groups maintained similar expression levels and patterns to the earlier timepoints. All injured sites had extensive inflammation-related differential gene expression, characterized by ≥ 24 upregulated and 2 or 3 downregulated inflammatory genes, and ≥21 upregulated with 2 downregulated immune genes. All injured sites had an increase in inflammatory and immune expression from 8WK to 16WK, with many genes becoming differentially expressed as shown in Fig. 2A and B. However, the level of expression for most differentially expressed inflammatory and immune genes was lower at 16WK than at 2WK. Some changes in differential expression between the 2WK and 8WK time points were maintained at 16WK. *Aim2* and *Msr1* continued to lack significant differential expression at 16WK, and *Mmp12* continued to be highly expressed at the stab wound and implant sites.

Neural health differentially expressed genes were mostly down-regulated across the injured sites, with 2–6 genes upregulated and 6 to 10 genes downregulated in the neural health category. The craniotomy-only site had many neural health genes differentially expressed that were significant at 2WK and not significant at 8WK. The 16WK implant site also had several genes become differently expressed that were not significantly expressed at 8WK. The stab wound had a similar number of genes differentially expressed at 16WK as there were at 8WK, but some lost significance while others gained significance. Genes related to myelination and oligodendrocyte function became differentially expressed at the 16WK craniotomy-only site with the stab wound site losing significant expression of *Mbp*, and *Olig2* (Fig. 2F).

The injured sites had mixed differential expressions of oxidative stress and apoptotic genes with each group having 4 or 5 upregulated and 4 to 6 downregulated genes in the oxidative stress category, and 2 or 3 upregulated and 3 downregulated apoptotic genes. Fig. 2D and E shows that the craniotomy-only site had many oxidative stress genes return to significance at levels similar to 2WK, with PTEN induced kinase 1 (*Pink1*) and superoxide dismutase 3 (*Sod3*) becoming uniquely differentially expressed. *Pink1* expression reacts to mitochondrial dysfunction and promotes mitophagy, and *Sod3* expression reacts to reactive oxygen species to remove them and reduce oxidative stress [58,59]. The stab wound and implant sites both maintained differential expression of most genes at 8WK with 2–3 genes either losing or gaining significance at both sites. In the homeostatic/growth category, a majority (≥18) of the genes were upregulated for all injured sites, but there were also several downregulated genes (≥6). The pattern of a large increase in differential expression from 8WK craniotomy-only site remains the same in the homeostatic/growth category (Fig. 2C). More homeostatic/growth genes were differentially expressed at the 16WK stab wound and implant sites than either the 2WK or 8WK timepoints. Overall, the craniotomy-only site had 75 upregulated and 30 down-regulated differentially expressed genes; the stab wound site had 99 upregulated and 23 downregulated differentially expressed genes; and the implanted site had 101 upregulated and 35 downregulated differentially expressed genes.

The uninjured site had 6 upregulated inflammatory differentially expressed genes with 3 downregulated inflammatory differentially expressed genes, 6 upregulated immune differentially expressed genes, and a single downregulated immune differentially expressed gene. *Fcrls, H2-T23, Itgax, Lc3b*, major histocompatibility complex, class I-related (*Mr1*), and *Spp1* all lost significance at 16WK from the 8WK timepoint, while coactosin-like F-actin binding protein 1 (*Cotl1*), olfactomedin-like 3 (*Olfml3*), proteasome 20S subunit beta 8 (*Psmb8*) became significantly upregulated, and *Atrn, Fyn, S1*pr*3* became significantly downregulated. There were 2 downregulated oxidative stress genes and 3 down-regulated apoptosis genes differentially expressed. *Fxn* and *Gsr* were significantly downregulated oxidative stress genes, and *Bax* was an additional significantly downregulated apoptotic gene when compared to 8WK. The uninjured site uniquely had no neural health genes and 8 upregulated with 1 downregulated homeostatic/growth genes. *Anxa1*, cluster of differentiation 274 (*Cd274*), fibronectin 1 (*Fn1*), heat shock protein family b (small) member 1 (*Hspb1*), and protein tyrosine phosphatase non-receptor type 6 (*Ptpn6*) were all differentially upregulated homeostatic/growth genes that became significant at the 16WK uninjured site and were not found at the 2WK and 8WK uninjured sites. At the uninjured site, a total of 20 differentially expressed genes were upregulated and 10 were downregulated.

### 4.4. Injury sites over time

**Fig. 9.**
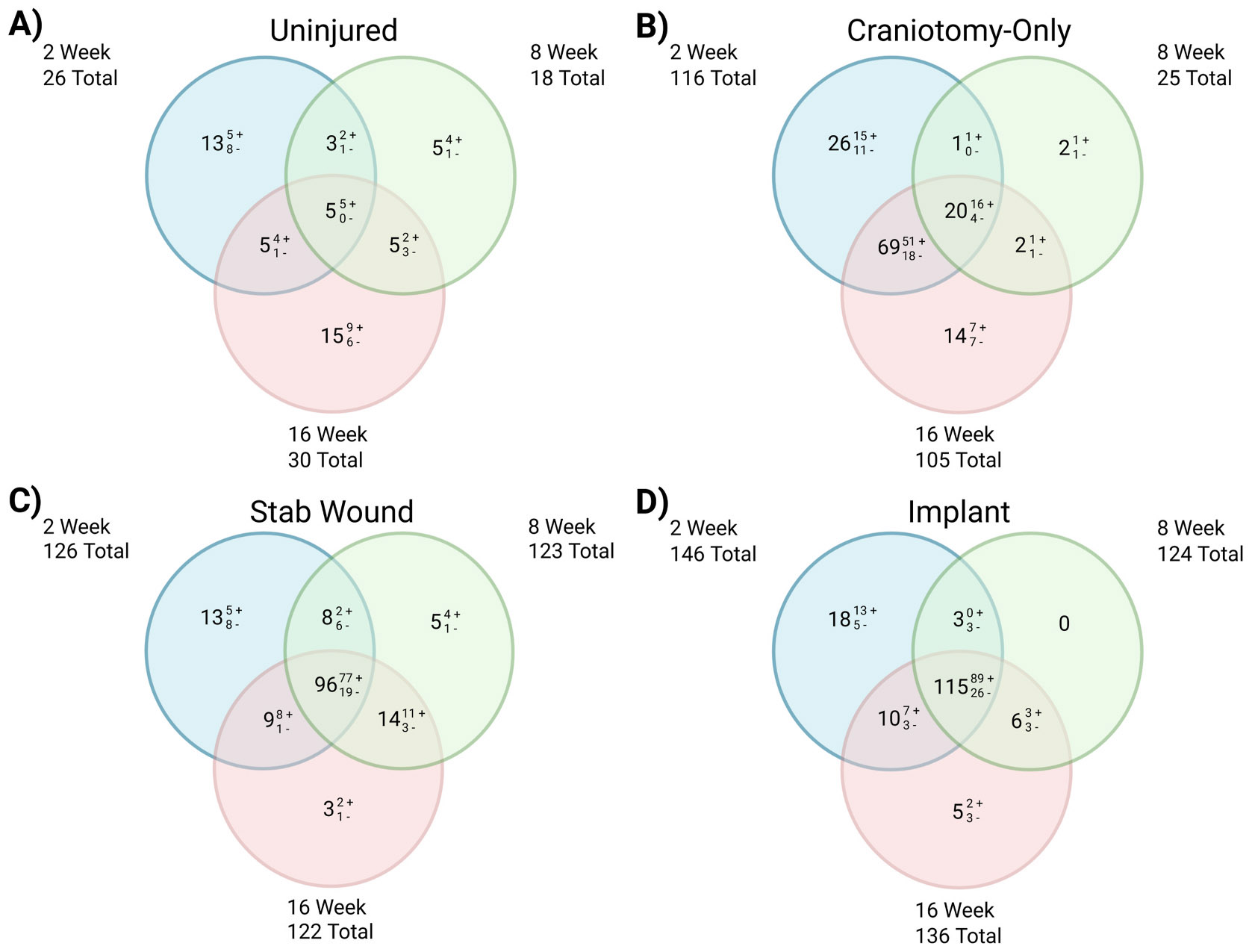
Venn diagrams of each injury site over time. Genes were included in the Venn diagram if their p_adj_ < 0.05. Overlapping regions indicate shared differentially expressed genes between time points. The numbers with the larger font size denote the overall number of differentially expressed genes with the smaller numbers being used to show up- or downregulated expression. The plus signs were attached to numbers of unregulated genes and minus signs were attached to numbers of downregulated genes. A-D) Venn diagrams of the injury sites’ differential expression over time and how expression at each time point is unique and overlaps. A) Uninjured site B) Craniotomy-only site C) Stab wound site D) Implant site.

Fig. 9 shows the temporal changes in the number of differentially expressed genes for each injury type (uninjured, craniotomy-only, stab wound, or implant). At the uninjured site (Fig. 9A and. 2) and 5 of the 51 (~10 %) differentially expressed genes were maintained across the duration of the study. All genes that continued to be differentially expressed at all time points were upregulated, with 4 out of 5 being inflammatory and immune-related genes. At most, ~31 % of the phenotype is conserved in the uninjured sites from 2WK to 8WK with 7 upregulated and 1 downregulated gene, and 55 % of the phenotype was conserved from 8WK to 16WK post-surgery with 7 upregulated and 3 downregulated genes. Of the 8 conserved differentially expressed genes from 2WK to 8WK, there were 7 (6 upregulated, 1 downregulated) from the inflammatory and immune categories. The differentially expressed genes conserved from 8WK to 16WK had a more varied breakdown, with 5 (4 upregulated, 1 downregulated) inflammatory and immune genes, 3 upregulated homeostatic/growth genes, and 2 downregulated apoptotic genes. All overlapping genes had similar log2-fold changes, indicating similarity in amount of expression.

At the craniotomy-only site (Fig. 9B and. 2) and 20 (16 upregulated, 4 downregulated) of the 134 (~15 %) differentially expressed genes were maintained across the duration of the study. Differentially expressed genes were conserved primarily in inflammatory and immune-related processes, with fewer homeostatic/growth genes conserved, and few genes conserved across time points from the other functional categories. From 2WK to 8WK post-injury, 21 (17 upregulated, 4 downregulated) of 116 (~18 %) genes continued to be differentially expressed. Further, the total number of differentially expressed genes decreased from 116 to 25 genes, with 4 (2 upregulated, 2 downregulated) newly significantly differentially expressed genes. From 8WK to 16WK post-injury, 22 (17 unregulated, 5 downregulated) of 25 (~88 %) differentially expressed genes remained differentially expressed. An additional 83 (58 upregulated, 25 downregulated) genes were differentially expressed at the craniotomy-only site at 16WK compared to 8WK post-injury. Notably, 69 (51 upregulated, 18 downregulated) of the 83 genes were previously differentially expressed (at 2 weeks post-injury), and 14 (7 upregulated, 7 downregulated) genes were differentially expressed solely at the 16WK post-injury timepoint. Fig. 2 shows all functional categories had most differentially expressed genes at 2WK return at 16WK, even in the absence of significant differential expression at 8WK.

At the stab wound site (Fig. 9C and. 2) and 96 (77 upregulated, 19 downregulated) of the 148 (~65 %) differentially expressed genes maintained significance across the duration of the study. While a substantial number of differentially expressed genes were conserved across all functional categories, those in the inflammatory and immune functional categories maintained the highest proportion of significant genes across time points. From 2WK to 8WK post-injury, 104 (79 upregulated, 25 downregulated) of the 126 (~83 %) differentially expressed genes were conserved. An additional 19 genes (15 upregulated, 4 down-regulated) were newly differentially expressed at 8WK, most belonging to the homeostatic/growth category. From 8WK to 16WK post-injury, 110 (88 upregulated, 22 downregulated) of 123 (~88 %) differentially expressed genes remained differentially expressed. An additional 12 (10 upregulated, 2 downregulated) genes were differentially expressed at the stab wound site at 16WK compared to 8WK post-injury, most belonging to the inflammatory and immune categories. Notably, 9 (8 upregulated, 1 downregulated) of the 12 additional genes were previously differentially expressed (at 2 weeks post-injury), and 3 (2 upregulated, 1 downregulated) genes were solely differentially expressed at the 16-week post-injury timepoint.

At the IME implant site (Fig. 9D and. 2) and 115 (89 upregulated, 26 downregulated) of the 159 (~72 %) differentially expressed genes were maintained across the duration of the study. Though many differentially expressed genes were conserved across all functional categories, those in the inflammatory, immune, and homeostatic/growth functional categories maintained the highest proportion of significant genes across time points. From 2WK to 8WK post-injury, 118 (89 upregulated, 29 down-regulated) of the 146 (~80 %) differentially expressed genes were conserved. An additional 6 genes (3 upregulated, 3 downregulated) were newly differentially expressed at 8WK, which belonged to the inflammatory (2 upregulated), oxidative stress (1 upregulated, 1 downregulated), apoptosis (1 downregulated), and homeostatic/growth (1 downregulated) categories. From 8WK to 16WK post-injury, 121 (92 upregulated, 29 downregulated) of 124 (~97 %) differentially expressed genes remained differentially expressed. An additional 15 (9 upregulated, 6 downregulated) genes were differentially expressed at the IME implant site at 16WK compared to 8WK post-injury, which were concentrated in the homeostatic/growth category (7 genes), while the inflammatory, immune, oxidative stress, and neural health categories each had 2 newly differentially expressed genes. Notably, 10 (7 upregulated, 3 downregulated) of the 15 additional genes were previously differentially expressed (at 2WK post-injury), and 5 (2 upregulated, 3 downregulated) genes were solely differentially expressed at the 16WK post-injury time point.

### 4.5. Pathway and gene ontology expression

**Fig. 10.**
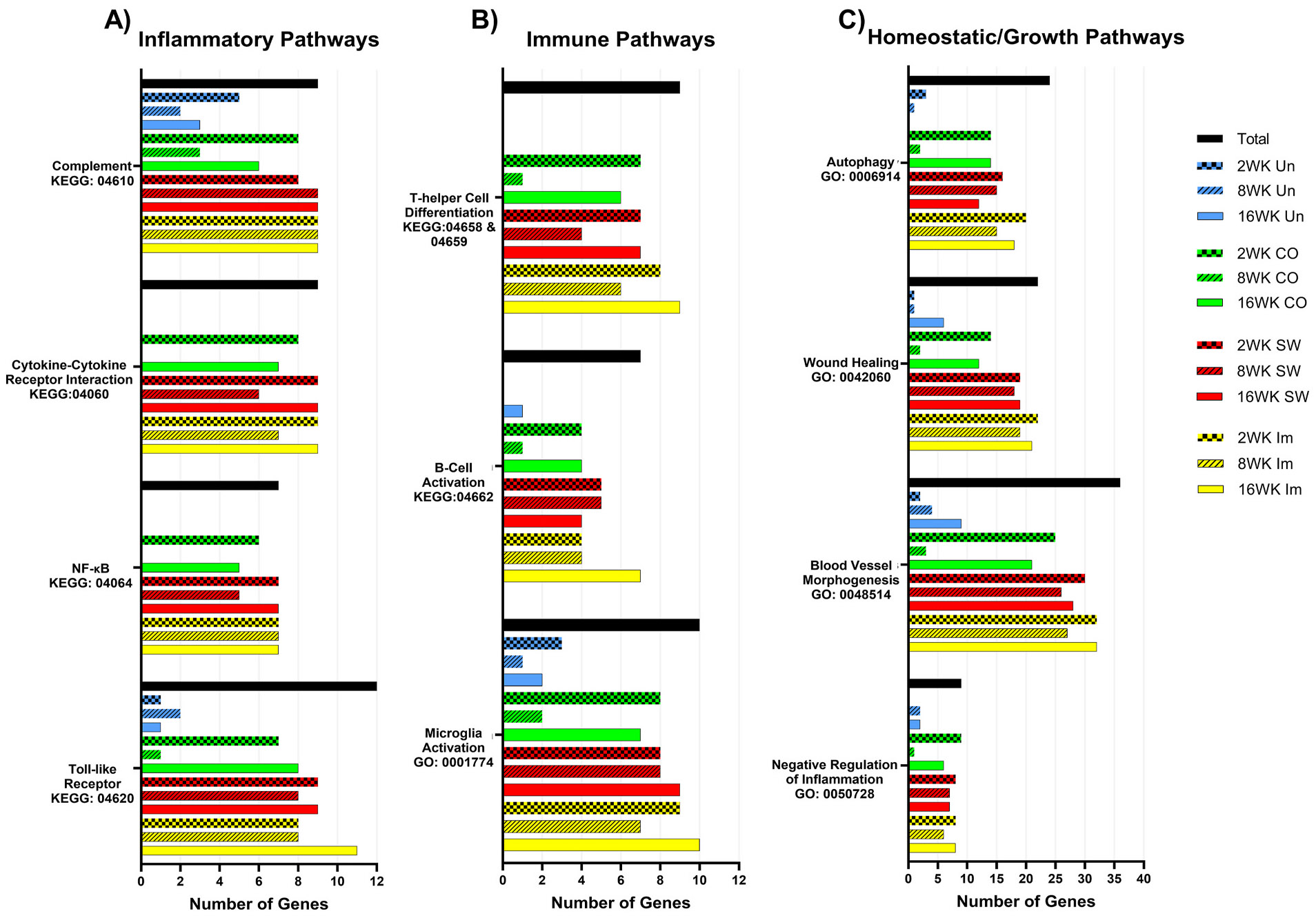
Bar plots of the counts of genes significantly expressed for each injury group in selected pathways and GO terms. Pathways and GO terms are grouped by functional category. The total bar is the total number of genes in the panel that were represented in each pathway. including non-significant genes. The bars for each injury group are the number of significant genes expressed. Colors were used to denote the injury type with yellow indicating implanted, red indicating stab wound, green indicating craniotomy-only, and blue indicating uninjured. Patterns were used to denote the timepoints where the 2WK timepoint had a checkered pattern, the 8WK timepoint had a diagonal pattern, and the 16WK timepoint had no pattern. A-C) Bar plots showing the significantly expressed gene counts for certain pathways and GO terms grouped by functional categories. A) Inflammatory pathways B) Immune pathways C) Homeostatic/growth pathways.

Pathway and GO term analysis revealed several biological processes in the inflammatory, immune, and homeostatic/growth categories that were enriched across most injured sites and time points, based on the proportion of differentially expressed genes from our targeted panel present in these pathways (Fig. 10). Although statistical significance is limited by the smaller gene set size, the consistent patterns observed across comparisons suggest biological relevance. Nearly all complement pathway (KEGG: 04610, *e.g. C3, C4a*) genes represented in our panel were differentially expressed in the stab wound and implant groups at all time points. In contrast, the craniotomy-only and uninjured sites exhibited a peak in the number of differentially expressed complement pathway genes at 2WK, which decreased at 8WK, and subsequently recovered by 16WK. Nearly all genes from our panel involved in the cytokine-cytokine receptor interaction (KEGG: 04060, *e.g.*, c-c motif chemokine ligand 5 (*Ccl5*), interleukin 1 beta (*Il1b*)) showed differential expression in the stab wound and implant site groups across time points, though fewer were differentially expressed at 8WK. In the craniotomy- only group, the number of significantly differentially expressed genes approaches that of the stab wound and implant site groups at 2WK and 16WK. No genes from the cytokine-cytokine receptor interaction pathway were significantly differentially expressed in the 8WK craniotomy-only group or in the uninjured group at any time point. The implant group showed differential expression for all genes in the NF-κB pathway (KEGG: 04064, *e.g.* phospholipase c gamma 2 (*Plcg2*), RELA proto-oncogene, NF-κB subunit (*Rela*)) at all experimental time points. The stab wound group exhibited a similar pattern, though there were fewer differentially expressed genes in the NF-κB pathway at 8WK. In the craniotomy-only group, several genes related to the pathway were differentially expressed at 2WK and 16WK. No NF-κB pathway genes were significantly differentially expressed in the 8WK craniotomy-only group or in the uninjured group at any time point. The toll-like receptor (TLR) pathway (KEGG: 04620, *e.g.* cluster of differentiation 14 (*Cd14*), *Spp1*) showed a high number of differentially expressed genes in the implant and stab wound groups across all time points. In the implant group, the number of differentially expressed TLR pathway genes was similar at 2WK and 8WK, with an increase at 16WK. The stab wound group maintained a relatively consistent number of differentially expressed genes over time. In the craniotomy-only group, the number of significantly differentially expressed genes decreased from 2WK to 8WK, then increased at 16WK to a level higher than at 2WK. The uninjured group had 1 to 2 TLR pathway genes significantly differentially expressed at any time point.

Pathways related to T-helper (Th) cell differentiation (KEGG: 04658 and KEGG: 04659, *e.g.*, cluster of differentiation 3 epsilon subunit of t-cell receptor complex (*Cd3e*), cluster of differentiation 4 (*Cd4*)) contained the same genes from our panel and were therefore analyzed together. In the implant group, the number of differentially expressed genes related to Th-cell differentiation decreased from 2WK to 8WK, then increased at 16WK. Both the stab wound and craniotomy-only groups showed similar trends, with reductions in differentially expressed genes related to Th-cell differentiation at 8WK and subsequent increases at 16WK. In contrast, no genes related to Th cell differentiation from the panel demonstrated significant differential expression in the uninjured group at any time point. Genes related to B-cell activation (KEGG: 04662, *e.g.*, B-cell linker (*Blnk*), *Plcg2*) were all significantly differentially expressed in the implant group at 16WK. At 2WK and 8WK, the implant group had similar numbers of differentially expressed B-cell activation genes to the stab wound group, as well as to the craniotomy-only group at 2 and 16 weeks. The craniotomy-only group at 8WK and the uninjured group at 16WK each exhibited one differentially expressed B-cell activation gene. A substantial number of genes associated with microglia activation (GO:0001774, *e.g.* integrin subunit alpha M (*Itgam*), complement C1q chain (*C1qa*)) genes were differentially expressed across time points in all injured groups, except for the 8WK craniotomy-only site, which had two differentially expressed genes. The uninjured group showed significant differential expression of three genes related to microglia activation at 2WK, and one or two at 8WK and 16WK.

Genes associated with autophagy (GO:0006914, *e.g.*, beclin 1 (*Becn1*), unc-51 like autophagy activating kinase 1 (*Ulk1*)) were highly represented among differentially expressed genes in the implant, stab wound, and 2WK and 16WK craniotomy-only groups. The implant group demonstrated the highest overall number of differentially expressed autophagy genes, while the stab wound group showed a consistent decline over time. With the craniotomy-only group, similar numbers of genes were differentially expressed at 2WK and 16WK, while few were detected at 8WK. The uninjured group had three or fewer differentially expressed autophagy-related genes at any time point. Genes involved in wound healing (GO:0042060, *e.g. Anxa1, Fn1*) were differentially expressed in similar numbers in both the implant and stab wound groups across time points, with a slightly higher number at the implant site. The craniotomy-only group had a moderate number of differentially expressed genes at 2WK and 16WK, but only a few differentially expressed genes at 8WK. In the uninjured group, one wound healing- related gene was differentially expressed at both 2WK and 8WK, which increased to 6 at 16WK. Many (>20) genes related to blood vessel morphogenesis (GO:0048514, *e.g. Cdh5, Hspb1*) were differentially expressed at each of the injured sites, except for the 8WK craniotomy-only site. At each time point, more blood vessel morphogenesis genes were differentially expressed in the implant group than the stab wound group, but both injury profiles exhibited similar patterns across time points. The craniotomy-only group at 2WK and 16WK showed a moderate number of differentially expressed genes from our panel in the blood vessel morphogenesis pathway, while at 8WK had only a few significantly expressed genes. The uninjured group uniquely demonstrated an increase in the number of significant blood vessel morphogenesis over time. Genes associated with the negative regulation of inflammation (GO:0050728, *e.g. Cd44, Ptpn6*) were highly expressed across injured sites except for the craniotomy-only group at 8WK. The implant sites showed a slight decrease in the number of differentially expressed genes at 8WK, with the count returning to the 2WK level by 16WK. The stab wound group maintained a consistent number of genes expressed across time points. The craniotomy-only group at 2WK had the largest number of differentially expressed genes related to negative inflammatory regulation, followed by a marked reduction at 8WK and a modest recovery at 16WK. The uninjured group at both 8WK and 16WK exhibited 2 significantly differentially expressed genes related to negative inflammatory regulation.

## 5. Discussion

In this study, we investigated the injury profiles of the component injuries that are required to perform an IME implantation to understand how they could be contributing to the overall neuroinflammatory response. The injuries resulting from craniotomy-only, stab wound, and implant surgical procedures were studied alongside an uninjured site in the same brain to evaluate more distant effects of the injuries. Tran-scriptomic analyses compared injured tissue to naïve controls to generate a broad picture of molecular mechanisms that were impacted around the sites of injury. The neuroinflammatory expression profiles of component injuries of IME implantation indicated that the craniotomy-only, stab wound, and implant injuries exhibited highly similar expression patterns. At each time point, the component injuries share at least 86 % of significantly differentially expressed genes with the implant site. Of the differentially expressed genes at the implant site, at least 76 % were shared with the component injuries, with the 8WK craniotomy-only group being the exception. All uninjured, craniotomy- only, and stab wound groups had at least 70 % of their significantly differentially expressed genes shared with the implant site. The high overlap indicates that the component injuries elicited similar physiological responses at both acute and chronic time points. The similarity in physiological response was correlated through a PCA (Supplementary Fig. S2) which had most samples clustered in a single region. The PCA also showed the difference in expression from the naive samples increased as severity of injury increased. Except for a single gene at each time point, all shared differentially expressed genes showed the same direction of regulation across groups, indicating that the underlying pathways were uniformly driven toward activation or suppression, rather than toward opposing responses in different injury types. Although the number and log2-fold change of significant differentially expressed genes differed greatly between the 8WK craniotomy-only and the 8WK implant sites relative to the naïve control, no differentially expressed genes were identified in a direct comparison of the two groups after Benjamini-Hochberg correction.

### 5.1. Inflammation and gene expression patterns

Across time points (excluding the 8WK craniotomy-only group), the number of differentially expressed genes related to inflammation, immune cell presence, and oxidative stress remained consistent, varying by no more than 4 genes per injury profile. The upregulation of most differentially expressed genes across injured groups in the inflammatory category indicates upregulation of complement, TLR, and NF-κB pathways, alongside cytokine release markers. The upregulated pathways associated with inflammatory genes indicate that many proin-flammatory processes are occurring in the tissue across the injured groups [34,41]. The complement system and cytokines act as key initiators for the inflammatory and the immune response [60-63]. Fig. 10 shows that a majority of genes represented in our panel for both pathways were differentially expressed across injured groups and timepoints. The expression of the complement pathway and cytokine-cytokine receptor interaction pathway is similarly found in other transcriptomic analyses of the IME injury site [33,41,64]. Genes such as *C3* and *C4a* are the most upregulated genes in the complement pathway and act as key factors in the downstream activation of innate immune cells [41]. *Ccl5* and *Il1b* are highly upregulated cytokine release markers that function to increase inflammation and recruit immune cells [33,65]. The upregulation of the complement pathway and cytokine release markers for most injured sites across time points indicates continued signaling of damage-associated molecular patterns (DAMPs) and, possibly, pathogen-associated molecular patterns (PAMPs) [62,63,66]. The data indicates that the implant site is the most affected by DAMPs due to it having the highest amount of expression in both the complement and cytokine pathways at every time point (Supplemental Fig. S3). The continuous activation of DAMPs is likely due to the presence of the implant causing continuous damage through micromotion or continued activation of immune cells [13,67,68]. Additionally , TLR proteins can be activated by PAMPs and DAMPs, and are responsible for the activation of inflammatory pathways and immune cells [66,69,70]. Over half of the genes related to TLR activation were consistently upregulated for the injured groups across time points. *Cd14,* a coreceptor for TLR4, and *Spp1,* a product of TLR signaling involved in immune cell recruitment, were highly upregulated, while *Mapk10*, a product of TLR signaling involved in apoptosis, was downregulated among injured groups and across time [71-73]. Bedell et al. analyzed the inflammatory response at the IME site at acute timepoints up to 2WK, and found similar activation patterns of the TLR pathway-associated genes, such as *Cd14, Itgam, and Casp8* at the 2WK time point [33]. The NF-κB pathway can be triggered by several factors, including cytokines and TLRs, and has a crucial role in recruiting the innate and adaptive immune responses, with additional roles in regulating inflammation [74,75]. Genes such as *Cd14* and *Tnfrsf1a*, a tumor necrosis factor alpha binding receptor that activates the NF-κB pathway, are significantly upregulated at all injured sites across timepoints, indicating activation of the NF-κB pathway as a factor in all injury conditions [71,76]. Notably, all genes expressed at each injured site in the NF-κB pathway had a small reduction in expression over time (Fig. 2, Supplemental Fig. S3). The consistent activation of the NF-κB and TLR pathways over time at the implant sites may be causing the glial scar components, such as astrocytes and microglia, to remain in a state of activation from acute to chronic timepoints [77,78]. The activation of inflammatory and immune pathways at even the craniotomy-only and stab wound sites across multiple time points highlights the contributions of the component iatrogenic injuries to the formation of the glial scar and an environment that promotes neurodegeneration.

Immune-related differentially expressed genes indicated increased T- helper cell differentiation, and markers for B-cell and microglia activation. The significantly upregulated immune cells are associated with proinflammatory responses and indicate that they are facilitating the prolonged inflammation experienced by each injury [79,80]. T-helper (Th) cells act as regulators of inflammatory processes such as cytokine production and immune cell recruitment [81]. Th-1 and Th-17 T-cells act as primarily proinflammatory stimulators where Th-2 cells have mixed anti- and pro-inflammatory effects [81,82]. Our custom gene expression panel cannot differentiate between types of Th-cell differentiation, so it is unclear which types were expressed at the injured sites. However, the presence of Th-cells at the injured sites is inferred through upregulation of markers such as *Cd4*, a marker for Th-cell presence, and *Cd3e*, a key factor in T-cell activation, across injury groups and most time points, with *Cd3e* losing significance at 8WK but returning at 16WK (Fig. 2) [82,83]. B cells are adaptive immune cells and act as directors and regulators of the immune and inflammatory response through cytokine, antigen, and antibody generation [84,85]. Fig. 10 shows that B cells are likely still active at chronic timepoints with genes such as *Blnk*, a key factor in B-cell differentiation, and *Ptpn6*, a regulator of B-cell receptor binding, significantly upregulated from 2WK to 16WK at all injured sites (Fig. 2) [86,87]. The continued presence of B cells in the brain may contribute to neurodegeneration and the loss of a healthy synaptic environment around the implant sites. B cells have been shown to be a primary facilitator in several neurodegenerative diseases acting as an amplifier for pro-inflammatory processes [88-90], suggesting an important role in neurodegeneration. Microglia have a dual role in the brain, contributing to maintenance of a healthy synaptic environment while also serving as the primary innate immune defense [70,77]. Consistent enrichment of activation markers such as *Itgam*, a receptor on microglia involved in phagocytosis, and *C1qa*, an activator of the complement pathway produced by microglia, across time points (Fig. 2) indicates that the microglia around injured sites are remaining in a proinflammatory state and are unable to perform their other function of modulating a healthy synaptic environment [91]. Microglia activation has also been found in several other IME transcriptomic studies, with proinflammatory microglial markers found at acute time points [33,35, 64]. A transcriptomic study by Joseph et al. (2021) suggests that elevated expression of *Hmox1* and *Tyrobp,* which can be associated with homeostatic microglia, at chronic time points indicates that activated microglia eventually return to a homeostatic phenotype [64]. In contrast, the results of our study show a strong presence of inflammation alongside several proinflammatory microglial markers, presenting a picture that the primary microglia phenotype at the 16WK chronic time point remains proinflammatory. The application of multiple injuries may have contributed to the discrepancies in microglia phenotype. Modulating injury-site microglia to restore their neuroprotective functions represents a potential strategy for reducing chronic inflammation at implant sites and in neurodegenerative diseases [92-95].

While oxidative stress genes were predominantly downregulated, a subset of three genes *Ccs, Gstp1, Hmox1* was consistently upregulated across almost all groups (Fig. 2). *Ccs* is associated with copper transport to superoxide dismutase 1 [96], *Gstp1* is related to glutathione oxidation [97], and *Hmox1* is associated with hypoxia. One transcriptomic study performed by Thompson et al. (2021) found significant expression of several oxidative stress genes at the implant site at acute timepoints but not the chronic timepoint (6 weeks) [35]. We found a similar drop in oxidative stress expression at the injury sites at 8WK, but at least several oxidative stress-related genes remained significant. The sustained upregulation of *Ccs, Gstp1*, and *Hmox1* across injury profiles and time points suggests that these genes are important to managing the chronic inflammatory environment. The prioritization of targeted detoxification processes may have resulted in the downregulation of other oxidative stress-associated processes [98]. Apoptosis-related genes displayed mixed expression (Fig. 3) with 2–3 genes being upregulated and 2–3 genes being downregulated. *Casp8* and *Tnfrsf1a* were consistently upregulated across experimental groups. The upregulation of *Casp8* and *Tnfrsf1a* is likely due to their dual role in apoptosis and inflammation, leading to their upregulation alongside other inflammatory markers [99-101]. Neuronal growth and health-related genes were generally downregulated (Fig. 2). Genes related to myelination and oligodendrocyte differentiation, such as *Mbp* and *Opalin*, were notably upregulated across most injury sites except the 8WK and 16WK implant groups. The pattern of expression indicates neuronal loss across injuries and a potential lack of axonal regrowth, but damaged neurons may be undergoing remyelination to restore synaptic function. The lack of upregulation for *Mbp* and *Opalin* in the 8WK and 16WK implant groups indicates a unique condition found at chronic implant sites that causes the inhibition of myelination and oligodendrocyte differentiation [36]. Autophagy-related genes (GO:0006914) largely showed down-regulation. Genes related to wound healing (GO:0042060), blood vessel morphogenesis (GO:0048514), and negative regulation of the inflammatory response (GO:0050728) were consistently upregulated across all groups. The biological processes related to the homeostatic/-growth functional category indicate the body’s struggle to remove cellular debris through the downregulation of autophagy [36]. Tissue restoration of the blood-brain barrier (BBB) occurs all the way up to 16WK post-surgery, as indicated by the wound healing and blood vessel morphogenesis upregulation [11]. The presence of several genes related to the negative regulation of the inflammatory response provides evidence that the body is trying to counter the proinflammatory response, but by 16WK post-surgery is still unsuccessful in mitigating inflammation [102]. Joseph et al. (2021) observed enrichment of wound healing and angiogenesis processes at chronic timepoints, and similarly concluded that the co-expression of the inflammatory processes, such as the complement pathway, indicates an incomplete healing of the tissue surrounding the implant [64].

The 8WK craniotomy group was unique in its relatively low number of significantly differentially expressed genes. However, upregulation was noted in genes associated with the complement cascade— *C1qa, C3*, and *C4a*; adaptive immune cells—*Fcrls* and basic leucine zipper ATF-like transcription factor 3 (*Batf3*); microglia—*Gpnmb* and *Olfml3*; and cell growth—*Itga6* and cyclin-dependent kinase 2 (*Cdk2*). In contrast, apoptosis-related genes—*Casp3* and *Mapk10*— were downregulated (Fig. 2 and Supplemental Table S1). The functional category classification of the differentially expressed genes indicates ongoing inflammation at the craniotomy site. Fig. 2 shows that the 8WK craniotomy-only group had a lower log2-fold change for genes in the inflammatory and immune functional groups compared to the other craniotomy-only time points. Mixed expression of anti-inflammatory, neural health, and glutathione reduction genes suggests that the upregulated healing processes have not been able to fully overcome the damage that occurred during the surgery. Overall, the craniotomy-only group at the 8WK time point exhibited a smaller number of significantly differentially expressed genes compared to the stab wound and implant sites, suggesting a dampened transcriptional response. The dampened response is further evidenced by Fig. 2 showing the log2-fold changes for each differentially expressed gene was lower than the same differentially expressed genes at the 8WK stab wound and 8WK implant sites. The results suggest that at the 8WK time point, there is an overall attenuation in inflammation occurring at the craniotomy-only sites compared to the stab wound and implant sites.

The decrease in differential expression at the craniotomy-only sites for the 8WK time point may be due to the craniotomy being the least severe of the 3 injuries assessed in this study, potentially allowing for partial recovery at an intermediate time point. Lagraoui et al. compared inflammatory responses evoked by controlled cortical impact (CCI) and a craniotomy and found the injury resulting from the craniotomy recovered at a faster rate and more completely than the more severe CCI [26]. For the implant, stab wound, and uninjured sites, the high similarity in expression profiles across time indicates a lack of recovery from inflammation by 16WK post-surgery. While the number of genes and average log2-fold change increased with the invasiveness of the injury, no genes were identified as significantly different when comparing injury groups directly. The lack of a significant recovery, combined with the lack of significant differences in gene expression between injury profiles, suggests that the acute injuries play a substantive role in the chronic inflammatory response observed surrounding IME implant sites.

The acute component injury groups and the implant group showed similar patterns of inflammation- and immune cell-related pathways at both the early 2WK and chronic 16 WK time points (Fig. 10), which covers a time frame that coincides with the observed decline in IME recording performance [8]. The particularly strong similarity in pathway activation patterns between the stab wound and implant across the study period suggests that the damage caused by IME insertion, and not solely chronic device presence, contributes to chronic neuroinflammation. Markers of gliosis and inflammation, including activated microglia, adaptive immune cell infiltration, and inflammatory upregulation, were present at both implant and stab wound sites across time points, suggesting that insertion alone can trigger reactive tissue changes that contribute to neuronal loss and degradation of neural recording performance [10,18,32]. Therefore, efforts to improve device design, such as by decreasing mechanical mismatch between the IME and the surrounding tissue are unlikely to be sufficient in mitigating the tissue response. Instead, strategies should also target inflammation arising from the implantation procedure and the acute component injuries. Several studies explored the effects of device insertion factors such as probe placement, insertion speed, probe size, and electrode sharpness, and found that these factors had significant differences in the tissue response to the insertion [103-106]. The potential for acute injuries to significantly impact the chronic inflammatory response validates the efforts from several labs focused on developing methods to reduce the impact of the implantation procedure on the targeted tissue [103,107-109].

While gene expression is important for understanding cellular processes and the direction they are set to operate, it is important to note that protein expression is the mechanism by which the body actuates functions. Recent studies have found that RNA expression and protein expression are not strongly correlated [110-113]. While the amount of correlation is not heavily disputed, there is some debate as to the degree to which protein expression can be inferred from RNA expression [110-113]. Some studies conclude that the low correlation means RNA expression can only loosely tell what functions the body is trying to perform with little determination on overall protein expression [110-112]. Koussounadis et al. analyzed the difference in the correlation distribution of non-differentially expressed and differentially expressed genes, and found that differentially expressed genes were more likely to have stronger correlations than non-differentially expressed genes. The authors argue that while the overall correlation tends to be low, the fact that differentially expressed genes had a significantly stronger correlation distribution indicates value in using RNA expression to infer protein expression [113]. A study performed by Edfors et al. (2016) analyzed cell lines grown from human tissue from several organs and found strong correlations between the protein and RNA counts in several tissue types. A key method was the normalization of protein and RNA counts based on cell counts, which they postulate is a key factor in quantifying the relationship between protein and RNA expression [114].

### 5.2. Uniquely differentially expressed genes as injury profile biomarkers

The genes that were uniquely expressed by one injury condition at any time point and those uniquely expressed across time points were further investigated (Supplemental Table S2) to assess their utility as injury-specific biomarkers. There were 27 genes uniquely differentially expressed by a single injury site group at the 2WK time point (Fig. 3A), 29 uniquely differentially expressed genes at the 8WK time point (Fig. 5A), and 23 uniquely differentially expressed genes at the 16WK time point (Fig. 7A). The magnitude of the log2-fold change for the uniquely differentially expressed genes ranged from 0.0968 (*Hif1a* in the implant group) to 5 (*Mmp12* in the implant group) at the 2WK time point, 0.123 (*Prnp* in the implant group) to 3.8 (*Il1b* in the implant group) at the 8WK time point, and 0.0667 (*Hif1a* in the implant group) to 0.707 (*Mbp* in the craniotomy-only group) at the 16WK time point. The identification of genes uniquely expressed in specific injury types and time points suggests their potential as biomarkers for distinguishing among injury profiles. In particular, *Mmp12, Itgax*, *Msr1, Aim2*, and *Cd274* are targets specific to the implant group at the acute 2WK time point; *Nr4a2, Opalin*, and *Mbp* are specific to the stab wound group at the subchronic 8WK time point; *Il1b, 1l2rg, Ccl5,* and *Icos* are specific to the implant group at the 8WK time point; *Mbp* is specific to the craniotomy- only group at the chronic 16WK time point; and *Icos* is specific to the implant group at the 16WK time point. Interpretation of individual differentially expressed genes should be made cautiously, as transcriptional changes in single genes may not be sufficient to infer pathway activation or functional outcomes without considering the broader expression context. However, in the craniotomy-only group, early downregulation of *Vps35* and *Pla2g6* and suggests that vesicular trafficking and lipid-remodeling deficits lead to chronic mitochondrial stress (*Pink1*), impaired autophagy (*Atg5*), and antioxidant decline (*Sod2, Sod3*). In the stab wound group, autophagy and signaling disruptions suggest direct axonal damage that evolves into prolonged demyelination and inflammatory signaling. In the implant group, early upregulation of neurotrophic and immune genes reflects an active tissue response around the device, progressing to long-term glial activation and oxidative metabolic shifts.

Among the 212 genes analyzed, thirteen were uniquely differentially expressed in one injury site condition and at a single time point (Fig. 2). *Pla2g6*, which plays a significant role in membrane phospholipid metabolism, signal transduction, and maintaining cellular homeostasis, was uniquely differentially expressed (downregulated) in the 2WK craniotomy-only group. *Ep300*, which can promote axonal regeneration after injury, was uniquely differentially expressed (downregulated) in the 2WK stab wound group [115]. The 2WK implant group uniquely expressed *Msr1* (upregulated), *Aim2* (upregulated), *Bid* (upregulated), *Mertk* (upregulated), and *Bdnf* (downregulated). *Msr1* plays a role in activating the NF-κB signaling pathway [45]. *Aim2* is involved in modulating the inflammatory response and release of pro-inflammatory cytokines [43]. *Bid* is involved in neuronal apoptosis [116]. *Mertk* is involved in clearing debris related to apoptosis after injury [117]. *Bdnf* is involved in neuronal regeneration [118]. *Fkbp5*, which is involved in inducing autophagy, was uniquely differentially expressed (upregulated) in the 8WK stab wound group [119]. The 16WK craniotomy-only group uniquely expressed *Pink1* (upregulated), *Tor1a* (downregulated), *Atg5* (downregulated), and *Sod3* (downregulated). *Pink1* encodes a neuroprotective protein that regulates mitochondrial function, oxidative stress, and apoptosis [120]. *Tor1a* is protective against oxidative stress [121]. *Atg5* is involved in neuronal autophagy, which is critical for maintaining neuron health [122]. *Sod3* scavenges reactive oxygen species to mitigate oxidative stress [59]. *Jun*, which plays a role in controlling inflammation and gliosis, was uniquely differentially expressed (upregulated) in the 16WK implant group [123]. There were no genes uniquely expressed in any injury group across all time points. Therefore, we are not able to assign particular genes as markers of particular injuries across all time points. However, *Cd83*, which encodes a protein that is present on activated antigen-presenting cells and is involved with the regulation and resolution of inflammation, was differentially expressed (downregulated) in only the 2WK and 8WK stab wound sites [124]. Therefore, *Cd83* may serve as a temporally-constrained marker of stab wound injuries for evaluating insertion methods to minimize damage [124].

### 5.3. Impact of IME implantation

The presence of the implant appears to exacerbate and prolong inflammation, even at uninjured regions 4 mm from the injury. Although the uninjured sites showed fewer differentially expressed genes, inflammatory markers were still significantly expressed (Figs. 3, 5 and 7). Distal inflammatory effects from traumatic brain injury (TBI) models have been established by several studies [125,126]. Our data indicates that even the implantation of a single-shank IME, which was previously thought to provoke only a localized tissue response, has distal inflammatory effects. Studies employing primarily IHC or similar histological analysis tools to characterize neuroinflammation following intracortical microelectrode implantation reported no significant difference between background fluorescence correlating to protein expression and the fluorescence at 500 μm from the point of injury [15,16,18]. However, the definition of “background” may significantly impact such findings, as some studies normalize fluorescent intensity to tissue 500 μm or more from the implant site, while others normalize to naïve animals [38,41,68,127-129]. Newer studies using gene expression analysis techniques have started to put the previously determined radius of inflammatory impact into question, with a few studies showing significant inflammatory expression as far as 1 mm from the point of injury [130,131]. The results of the present study show that prolonged inflammation persists even at distant uninjured sites, likely due to the implant serving as a constant irritant [68].

The study of the injury profiles was conducted using injuries applied to and tissue analyzed from four quadrants of the cortex, divided by the sagittal and coronal sutures, leading to tissue from both the motor cortex (anterior to bregma) and the somatosensory cortex (posterior to bregma). The regional differences in brain structure, vascular density, cellular composition, and immune responses could lead to differential responses to the injuries [132]. Additionally, positional differences of the implant can alter the mechanical strain profiles imparted onto tissue, which may also affect the tissue response [68,129,133]. To mitigate these differences and avoid biasing the results, the sites of the applied injuries were rotated such that at each time point, the tissue samples for each injury profile were from different quadrants. We further ensured that the regional differences did not bias our results by comparing the gene expression of tissue from the motor cortex to that of the somatosensory cortex in the naïve control tissue (Supplemental Fig. S1). There were no significant differences in baseline expression in the two tissue types. Similarly, in a spatial proteomics study involving implanted microelectrodes, Druschel et al. found that the regional differences from tissue samples taken from the motor and somatosensory cortex did not produce meaningfully different immune cell and neural cell protein profiles [110]. Therefore, differential gene expression in the experimental groups results from changes in gene expression related to the applied injuries, and not to the specific regions where the injuries were applied.

The findings of the current study are consistent with previous IHC studies showing that the implant gives rise to chronic neuro-inflammation. Potter et al. analyzed contralateral stab wounds and implants and found stab wounds recovered neuron populations over time, while implants led to persistent inflammation and neuron loss [11]. McConnell et al. (2009), using separate animals for stab wounds and implants, observed no effects from stab wounds by 16WK [18]. In contrast, the present study used a rotating placement of injuries and found prolonged inflammation with high overlap in differentially expressed genes between stab wound and implant sites at 2WK, 8WK, and 16WK time points. The conflict between these studies suggests that there is a mechanism by which the implant prolongs the inflammation at the injured sites and delays tissue recovery.

A plausible mechanism for widespread inflammation is BBB disruption. Several studies have confirmed an increase in BBB permeability, with both brain injuries and peripheral injuries leading to systemic effects on BBB integrity and vascular structure [134-138]. Therefore, inflammatory markers from the injury sites may be entering the blood vessels and disrupting the BBB in other areas, thereby promoting inflammation at distant locations. Research indicates that individuals with traumatic brain injuries (TBIs) have a higher risk of neurodegeneration and developing neurological diseases [139-141]. The worsening neurological outcomes have been theorized to occur due to the initial injury leading to persistent inflammation in both gray and white matter that creates a buildup of damage over time [141].

The discrepancy between recovery timelines for stab wounds in implant-free versus implant models demonstrates the effects of the persistent irritation from the implant. Prior studies report recovery from stab wounds within 30–60 days (4–8 weeks) after injury [142,143], while our findings suggest prolonged inflammation in the presence of the implant. The constant aggravation caused by differential strain applied at the implant-tissue interface due to mechanical mismatch and micromotion may be confounding the recovery of the acute injury sites (craniotomy-only and stab wound) [13,14]. Therefore, strategies to mitigate implant-induced inflammation may aid in improving recovery outcomes after IME implantation.

Though our study was the first to systematically investigate the transcriptomic response at the component injury sites, the implant group gene expression profiles are consistent with earlier studies from our lab and others studying IME-induced changes in gene expression. For example, upregulation of complement pathway genes was consistently detected at both acute and chronic time points, matching findings from earlier studies [33,41]. In addition, our data confirm increased differential expression of genes associated with astrocyte activation, a feature of the well-established neuroinflammatory response following neural device implantation [33,35]. Healing processes were also affected by the implant (Fig. 10), supporting prior observations that expression of genes involved in the healing process is altered by the implant process and the sustained IME presence [35,41]. Thompson et al. (2021) analyzed several genes related to oxidative stress and similarly found mixed up- and down-regulation at acute and more chronic timepoints [35]. Our results corroborate previous studies showing differential expression of genes related to microglia activation in response to IME implantation [33,35,41], providing further support for the important role of the innate immune response in the tissue response to implants. The chronic upregulation of inflammatory pathways for the implant group supports findings from earlier studies, including those employing flexible probes to minimize chronic damage induced by micromotion [35,41]. Taken together, these findings reinforce the notion that implant-induced neuroinflammation and repair processes are robust phenomena, persisting across experiments and device technologies. Future work should focus on minimizing the injury caused by the IME implant process, while continuing to improve neural interface design to better integrate with tissue.

## 6. Conclusion

In this study, the neuroinflammatory gene expression profiles for a craniotomy, stab wound, implant site, and uninjured site were studied in rats using a custom panel of 212 genes. Gene expression at 2-week, 8-week, and 16-week time points were compared to non-surgical naïve rats to determine how each injury’s expression profile changed from the acute to chronic time points. Through this study, we aimed to advance our understanding of whether and how acute injuries contribute to the acute and chronic tissue responses observed following intracortical microelectrode implant surgery.

The results showed that both the craniotomy-only and stab wound injury sites had high levels of neuroinflammatory gene expression at acute and chronic time points. Greater injury invasiveness was associated with more differentially expressed genes and larger log2-fold changes. Gene expression profiles remained largely consistent across injury types and time points, except for the craniotomy-only sites at 8 weeks. The consistency was indicated by a high degree of overlap in differentially expressed genes and direction of regulation among injury profiles that was maintained across the three time points. The extensive overlap suggests that the acute injuries (craniotomy and stab wound) play a major role in the neuroinflammatory response observed surrounding implanted intracortical microelectrodes. A substantial number of genes were differentially expressed at the uninjured sites, demonstrating the complexity of the tissue response and the broad reach of the effects of the acute injuries and chronic presence of IMEs. As a result, the specific contribution of each of the acute injuries to the composite response to IME implantation cannot be isolated. Gene expression at ostensibly uninjured sites indicates that the tissue impact of implanted devices extends farther into the brain than previously appreciated. The continuous presence of the IME in the brain may have promoted prolonged inflammation at the injury sites through constant BBB disruption. The possibility of distal neuroinflammatory effects creates uncertainty as to how multiple implants within the same brain affect each other. Whether this inflammation is additive or insignificant to implantation sites is important to understand due to complex human BMI systems requiring more than one implant to function.

In this study, chronic neuroinflammation resulting from the chronic presence of the implant acts as a confounding factor and limits the ability for a definitive determination of the role the acute injuries play in the tissue response. Further investigation of the expression profiles for the stab wound and craniotomy without the influence of chronic irritation from the implant would be valuable to isolate the contribution of each component injury to the inflammation around implanted devices. An enhanced understanding of acute injury mechanisms may inform strategies to reduce their contribution to the overall neuroinflammatory response. Minimizing acute damage through improved implantation techniques may ultimately extend the functional lifespan of intracortical microelectrode devices.

## Supplementary Material

1

2

## Data Availability

Data will be made available on request.

## Appendix A. Supplementary data

Supplementary data to this article can be found online at https://doi.org/10.1016/j.biomaterials.2025.123692.
