# Supplementary material for: Transcriptomic analysis of the physiological responses to injuries induced accompanying intracortical microelectrode implantation
Source: Biomaterials. Author manuscript; Available in PMC 2026 Jul 21. (PMC13386284; doi:10.1016/j.biomaterials.2025.123692)
Supplement: 1 [file NIHMS2182858-supplement-1.docx]

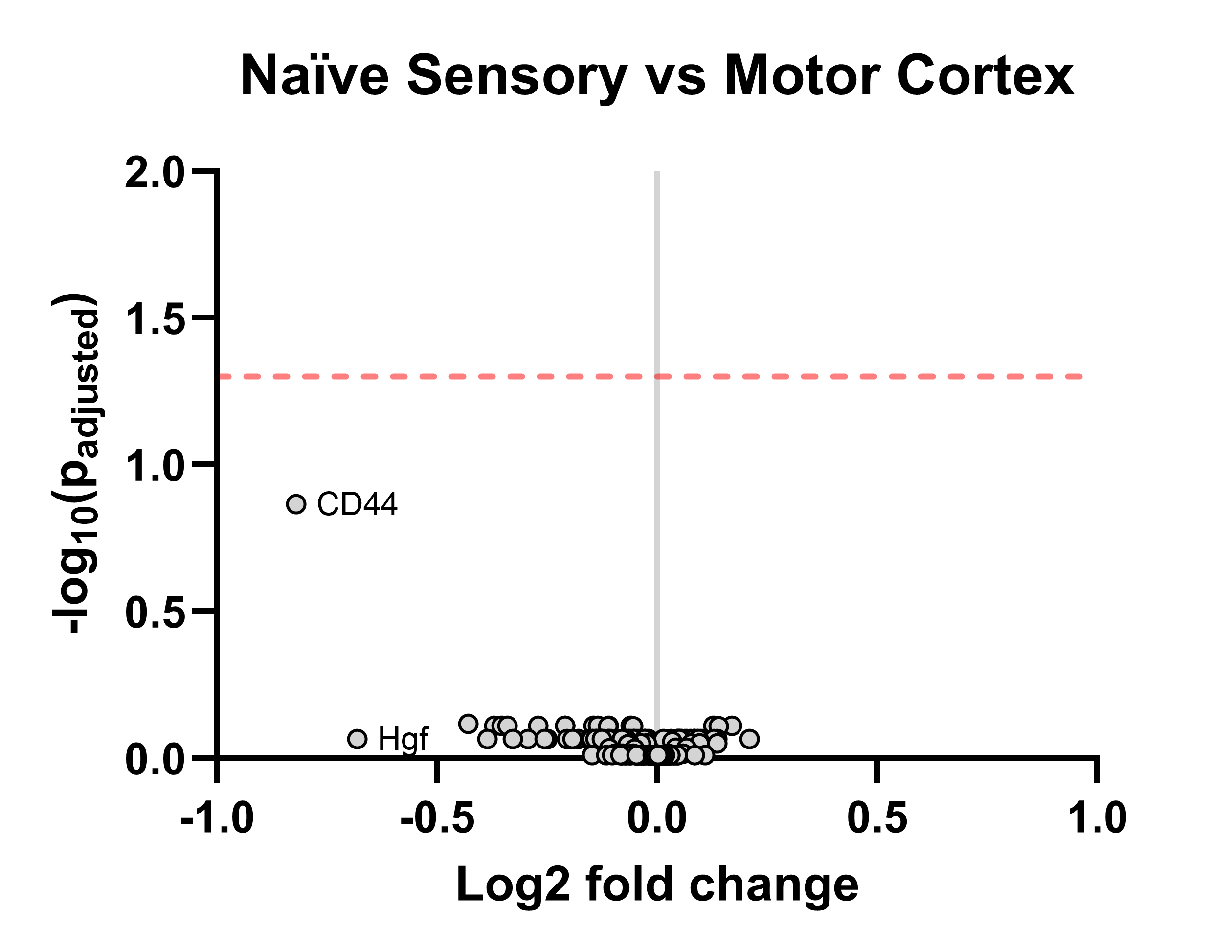


**Figure S1:** Volcano plot showing the differential expression of the naïve animals motor cortex when compared to the baseline of the somatosensory cortex. No genes were significantly expressed (p_adj_<0.05). The gray line marks the divide between up- and downregulated genes, with upregulated genes located to the right of the line and downregulated genes located to the left. The dotted red line marks the threshold (p_adj_<0.05) for genes to be considered significant.


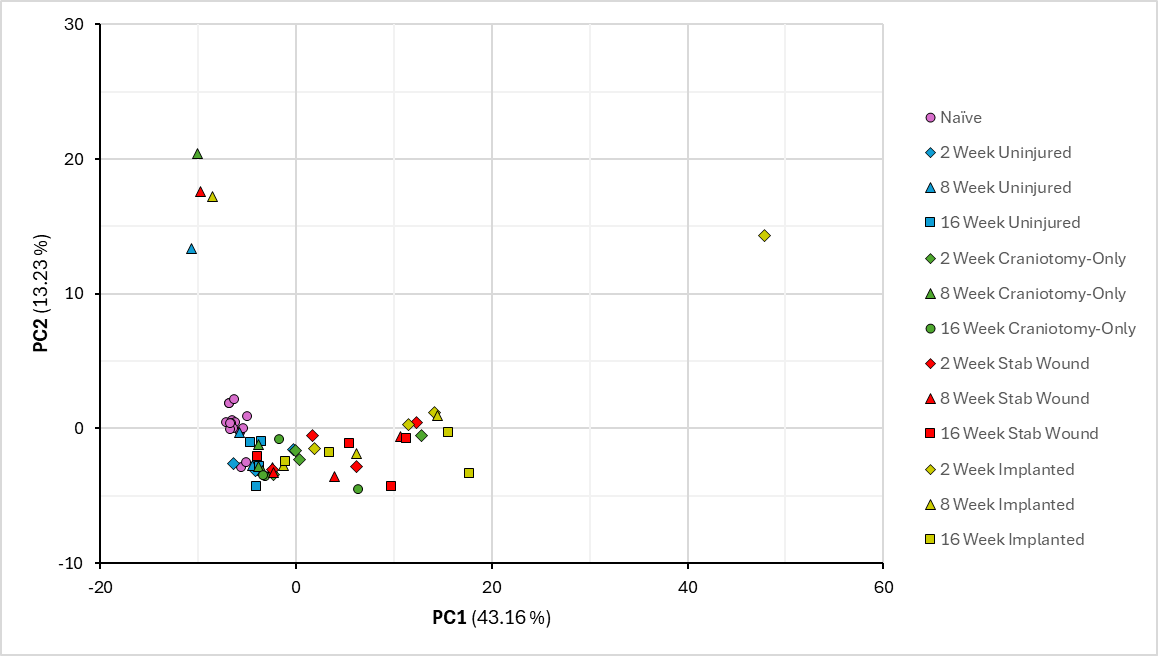


**Figure S2:** Scatter plot of the values of the first 2 principal components for each sample. The naive samples have the smallest grouping of values followed closely by the uninjured group. The samples from the injured groups experience increasing variability in values correlating alongside an increase in the severity of injury with the craniotomy-only samples having the least and the implant samples having the most. Several sample values were grouped separately from the rest of the samples which all correlated to a single animal in the 8WK timepoint group.

**Table S2:** Table of all unique genes significantly expressed for each injury group at each time point alongside their log2-fold changes and adjusted p-values. The genes at each time point are grouped by the injury profile in which they were uniquely expressed.


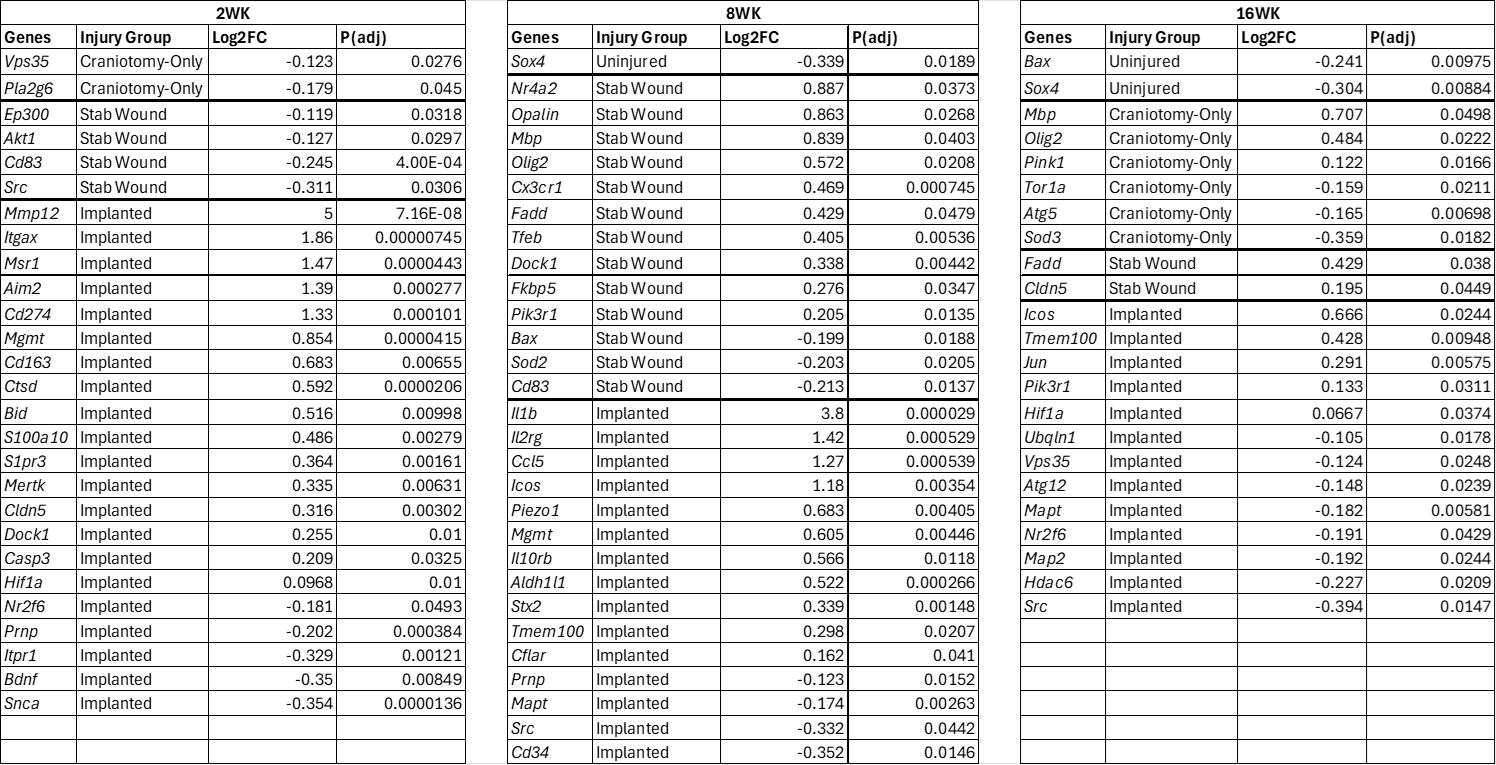


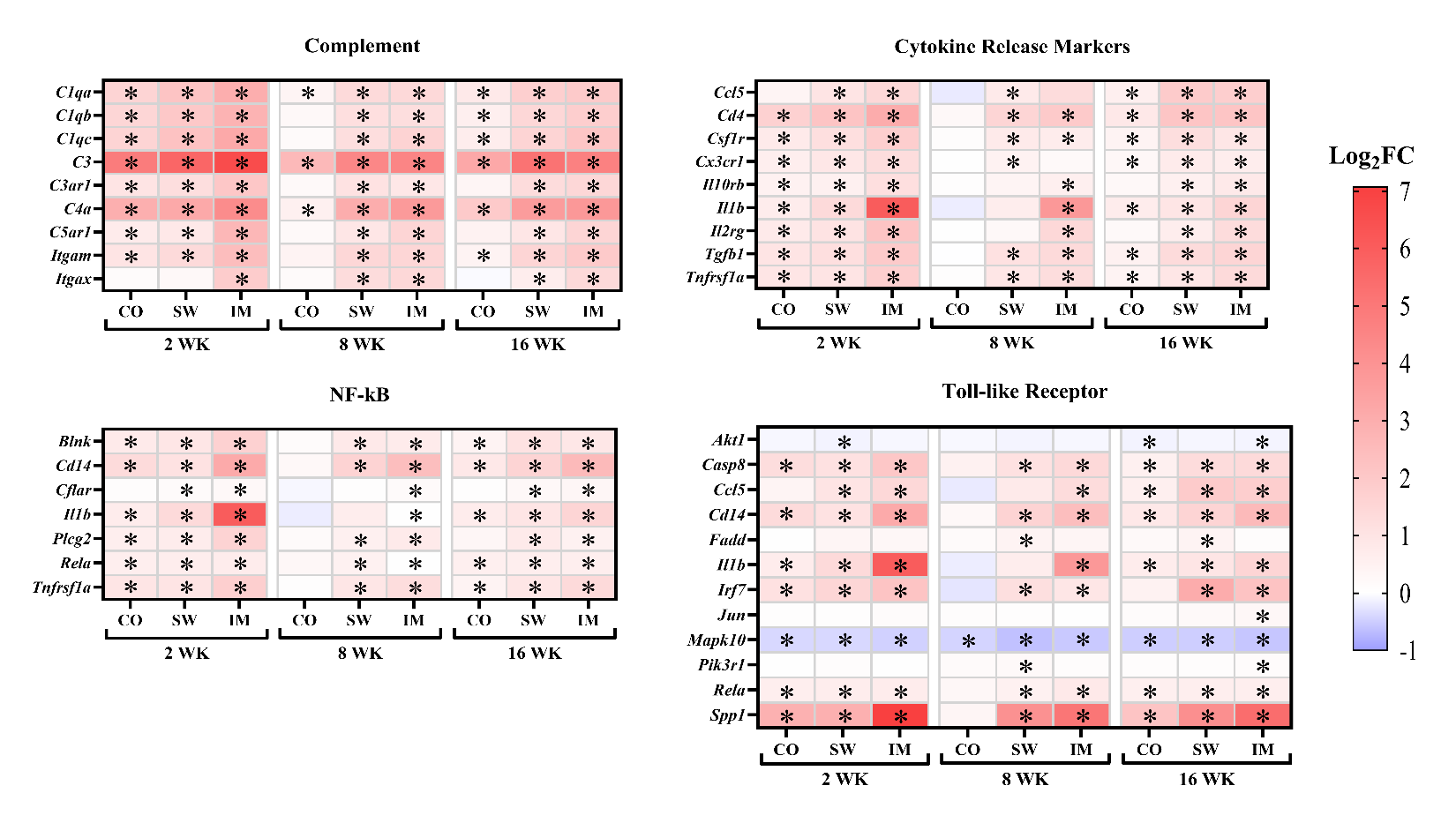


**Figure S3:** Heatmaps of differentially expressed genes in every inflammatory pathway. Red represents upregulation and blue represents downregulation compared to naïve controls. Genes were included if they were differentially expressed in at least 1 experimental group. Stars are located on where genes were significantly expressed (p_adj_<0.05).
